# Clustering single-cell multimodal omics data with jrSiCKLSNMF

**DOI:** 10.3389/fgene.2023.1179439

**Published:** 2023-06-09

**Authors:** Dorothy Ellis, Arkaprava Roy, Susmita Datta

**Affiliations:** Department of Biostatistics, University of Florida, Gainesville, FL, United States

**Keywords:** graph regularization, KL divergence, multimodal omics, multiplicative updates, scATAC-seq, scRNA-seq, sparsity

## Abstract

**Introduction:** The development of multimodal single-cell omics methods has enabled the collection of data across different omics modalities from the same set of single cells. Each omics modality provides unique information about cell type and function, so the ability to integrate data from different modalities can provide deeper insights into cellular functions. Often, single-cell omics data can prove challenging to model because of high dimensionality, sparsity, and technical noise.

**Methods:** We propose a novel multimodal data analysis method called **j**oint graph-**r**egularized **Si**ngle-**C**ell **K**ullback-**L**eibler **S**parse **N**on-negative **M**atrix **F**actorization (jrSiCKLSNMF, pronounced “junior sickles NMF”) that extracts latent factors shared across omics modalities within the same set of single cells.

**Results:** We compare our clustering algorithm to several existing methods on four sets of data simulated from third party software. We also apply our algorithm to a real set of cell line data.

**Discussion:** We show overwhelmingly better clustering performance than several existing methods on the simulated data. On a real multimodal omics dataset, we also find our method to produce scientifically accurate clustering results.

## 1 Introduction

Next-generation sequencing (NGS) technologies have enabled the extraction of large amounts of cellular information from biological tissues. These data are collectively known as omics and include metabolomics, transcriptomics, epigenomics, proteomics, and metagenomics. Within the last decade, the integration of multiple omics profiles has led to advances in precision medicine and the identification of underlying disease mechanisms (Reel et al., 2021). Furthermore, advances in single-cell sequencing technologies have enabled the extraction of omic profiles at the resolution of a single-cell (Tang et al., 2009; Buenrostro et al., 2015). Within the last half-decade, the extraction of multiple omics profiles from the same set of single cells has become possible (Stoeckius et al., 2017; Chen et al., 2019; Ma et al., 2020; Swanson et al., 2021). Lee et al. (2020) and Ogbeide et al. (2022) detail a wide variety of technologies currently available to collect data from multiple omics modalities from the same set of cells. The genome, transcriptome, and proteome are connected through the central dogma of molecular biology: DNA is transcribed to RNA, which is in turn translated to proteins (Li and Biggin, 2015). Costa Dos Santos et al. (2021) discuss an extension to the central dogma; in this updated version, the metabolome drives the flow of omics information through the cell. This updated version also includes the epigenome, which are biochemical modifications to DNA that affect structure and regulation of the genome (Park et al., 2016). These include histone modifications, chromatin accessibility, and DNA methylation. While omics data collected from the same cell are all inter-related, each modality still provides some unique information about that cell. Thus, the integration of these data across omics modalities can enable deeper insights into cellular functions than the analysis of each modality in isolation. Among these deeper insights is improved cell-type clustering. Expression of omics data varies among cell types, and this cellular heterogeneity is not captured in bulk data (Ellis et al., 2021). Accurately clustering cells can, for example, enable insights into and analysis of cell-type-specific responses to disease. Additionally, some omics modalities are more informative for differentiating between certain cell types than others; for example, in Hao et al. (2021), CD4^+^ cells and CD8^+^ cells had similar RNA expression profiles but had different protein expression profiles. Currently, there are only a few methods available to integrate count data across multiple single-cell omics modalities. Many of these methods require log(*x* + 1) normalization methods that can introduce bias into the transformed data by exaggerating the differences between 0 and low count observations (Townes et al., 2019; Elyanow et al., 2020). Most other methods also choose a fixed number of highly variable features on which to perform clustering; however, these highly variable features may not necessarily be the most informative for cell clustering and can leave out important information (Townes et al., 2019). Hence, we develop **j**oint graph-**r**egularized **Si**ngle-**C**ell **K**ullback-**L**eibler **S**parse **N**on-negative **M**atrix **F**actorization (jrSiCKLSNMF, pronounced “junior sickles NMF”) for the count-valued omics data within each modality while integrating across omics information in order to offer more accurate cell-type clustering. Through our method, we aim first to extract latent factors that are relevant to cell-type clustering and consequently enable convenient clustering on these latent factors. Secondly, we allow the visualization of cell type clusters by leveraging the data compression abilities of NMF. Non-negative matrix factorization has been used for various modern applications, including latent factor extraction, data compression, and clustering. Additionally, many NMF methods have already been applied to the analysis of omics data. These include Multi-NMF (Liu et al., 2013; Wang et al., 2015; Rappoport and Shamir, 2018), integrative NMF (Chalise and Fridley, 2017; Liu et al., 2020), and jNMF (Greene and Cunningham, 2009; Akata et al., 2011; Wang et al., 2015; Dai et al., 2020) for multi-omics data; NMF (Lee and Seung, 1999) and graph-regularized NMF (Cai et al., 2008; 2011; Elyanow et al., 2020) for single-modality omics data; SC-JNMF (Shiga et al., 2021) for different quantifications of scRNA-seq data measured on the same set of cells; and scAI (Jin et al., 2020), which, like our method, is for multimodal single-cell omics data. Some of these methods, including jNMF, Multi-NMF, and graph-regularized NMF, arose first from the fields of image processing and document classification.

Although our method can theoretically integrate any number of modalities of single-cell count-valued data collected from the same set of cells or any number of bulk assays collected from the same individual, we primarily focus on integrating single-cell RNA-sequencing (scRNA-seq) and single-cell assay for transposase-accessible chromatin using sequencing (scATAC-seq) data from the same set of single cells. Methods for collecting these data include sci-CAR (Cao et al., 2018), SNARE-seq (Chen et al., 2019), SHARE-seq (Ma et al., 2020). scRNA-seq allows for the detection and analysis of messenger RNAs (mRNAs) at a single-cell resolution. These data consist of count matrices where each column corresponds to a cell and each row to a gene (Haque et al., 2017). scATAC-seq identifies accessible regions (peaks) within the chromatin of a single-cell; the data consist of matrices of counts of nucleosome free region (NFR) fragments, where each column corresponds to a cell and each row corresponds to a given range of base pairs (Yan et al., 2020). Due to several challenges such as batch effects, technical noise, and sparsity, these data require extensive quality control, normalization, and batch effect correction before downstream analyses, including cell clustering and annotation, network analysis, and differential expression analysis, can proceed (Yan et al., 2020; Ellis et al., 2021).

In Section 2, we discuss the motivation for our model in detail, provide the loss function, and discuss the implementation. We discuss both the initialization of our matrix product approximation as well as the optimization of this product. “Joint” NMF methods share one of either feature matrix **W** or observation matrix **H** across different modalities of data or different individuals. For jrSiCKLSNMF, we share **H** across all omics modalities and treat it as a latent cell-specific factor matrix. To adjust for differences in quality and quantity of information across modalities, we use graph regularization on each modality *v*’s **W** ^*v*^ matrix. Elyanow et al. (2020), whose research also served as a primary motivation for this work, detail this approach of using graph regularization for the feature matrix **W** for single-modality scRNA-seq data. Because both modalities of these data are inherently sparse, we also include a sparsity constraint on **H** or, alternatively, a unit norm constraint on the L2 norm of the rows of **H** as detailed for single-modality data in Le Roux et al. (2015). Because we are integrating different types of count data, we use the Poisson Kullback-Leibler (KL) divergence across all modalities.

In Section 3, we compare our method with competing methods on simulated data. We also provide a real data example. While there are a multitude of methods currently available for integrating bulk omics data across modalities and also methods to integrate data from different single-cell populations measured on the same individual (Krassowski et al., 2020; Subramanian et al., 2020; Miao et al., 2021), there are only a few approaches for the integration of measurements from the same set of single cells. Some of these methods include Seurat v. 4 (Hao et al., 2021), BREM-SC (Wang et al., 2020), CiteFuse (Kim et al., 2020), scAI, and MOFA+ (Argelaguet et al., 2020). We briefly discuss these existing methods in Section 3.3 before comparing them to jrSiCKLSNMF in Section 3.4. Of these, only our method and BREM-SC take into account the count nature of both the scATAC-seq and scRNA-seq modalities; all other methods require some form of log normalization on the data. Coincidentally, BREM-SC, which assumes the data follow a Dirichlet-Multinomial distribution, was, after the four variations of jrSiCKLSNMF, the fifth highest performing method on simulated data with no introduced noise. Finally, in Section 4, we discuss potential extensions of jrSiCKLSNMF as well as its limitations.

## 2 Materials and methods

In general, all non-negative matrix factorization (NMF) models attempt to find a reduced rank latent representation, where the number of latent factors often is pre-specified (Lee and Seung, 1999). Among various uses of NMF, our method is, primarily, designed for clustering cell types by first extracting latent factors shared across omics modalities and then clustering these latent factors using any clustering method. We perform all analyses and coding in R (R Core Team, 2022). We also make extensive use of the Rcpp and RcppArmadillo packages from Eddelbuettel and François (2011) and Eddelbuettel and Sanderson (2014), respectively. In the next subsection, we introduce and develop our proposed joint NMF model based on the KL divergence with regularization and sparsity constraints.

### 2.1 Non-negative matrix factorization (NMF)

As detailed above, NMF algorithms approximate an observed, *M* features by *N* observations, non-negative data matrix **X** as the product of an *M* × *D* non-negative reduced-dimension feature matrix **W** and a *D* × *N* non-negative reduced-dimension observation matrix **H** such that

X≈WH, (1)

where *D* < min{*M*, *N*} is the rank of this approximation. Hence, NMF aims to produce a reduced rank approximation of the original non-negative data matrix **X**. For any *D* × *D* non-negative invertible matrix **Q**, we have **WQQ** ^−1^ **H** = **WH**. This implies that (**W**, **H**) and (**WQ**, **Q** ^**−1**^ **H**) lead to equivalent approximations. Because of this, **W** and **H** are not identifiable. The required conditions for identifiability complicate the computational steps, and there has been much work to determine sufficient identifiability criteria (Fu et al., 2018; 2019; Gillis and Rajkó, 2023). However, we can restrict the parameter space by applying different constraints on **W** and **H**. Specifically, we use a graph regularization constraint on **W** and propose two possible constraints on **H**. The first one is a sparsity constraint with a Frobenius norm penalty. The second constraint sets the L2 norm of the rows of **H** to 1. These two constraints are compared in simulations. These constraints, along with the non-negative constraints on **W** and **H**, though they do not by any means solve the identifiability issue, can help to mitigate it by reducing the possible solution space for **Q** Fu et al. (2019). Additionally, graph regularization constraints on the **W** matrix ensure the preservation of geometrical structures within the data. Both the sparsity constraint on **H** and the graph regularization constraint on **W** enforce sparsity, which is desirable due to sparsity in single-cell omics data (Cai et al., 2008; 2011; Kimura and Yoshida, 2011; Gillis, 2012; Peng et al., 2019; Zhou et al., 2021). Moreover, the unit L2 norm constraint on the rows of **H** enables us to avoid tuning the regularization parameter *λ*_*H*_ without sacrificing any accuracy in the clustering results for lower noise levels in our simulation study. The use of the L2 norm constraint also appears, for our real data example, to extract more meaningful factors in the Uniform Manifold Approximation and Projection (UMAP) (McInnes et al., 2018) plots. In order to approximate **X** as **WH**, the most common techniques are to minimize the square of the Frobenius norm of the difference between **X** and **WH** or to minimize the KL or Itakura-Saito (IS) divergence between the two matrices (Lee and Seung, 1999; Févotte et al., 2009). These methods are all special cases of the *β*-divergence, with *β* = 0, 1, 2 for the Frobenius norm, KL divergence, and IS divergence, respectively Févotte and Idier (2011). For our method, we minimize the loss based on the KL divergence between Poisson(**X**) and Poisson(**WH**) as in Elyanow et al. (2020). Even though **WH**, the approximation of **X** is of the same dimension, the data contained in **WH** are of lower resolution compared to the original matrix **X**. This data compression property of NMF can be helpful for data visualization on top of using the reduced dimensional matrix **H** generated by jrSiCKLSNMF algorithm for clustering.

### 2.2 Motivation for jrSiCKLSNMF

**FIGURE 1:**
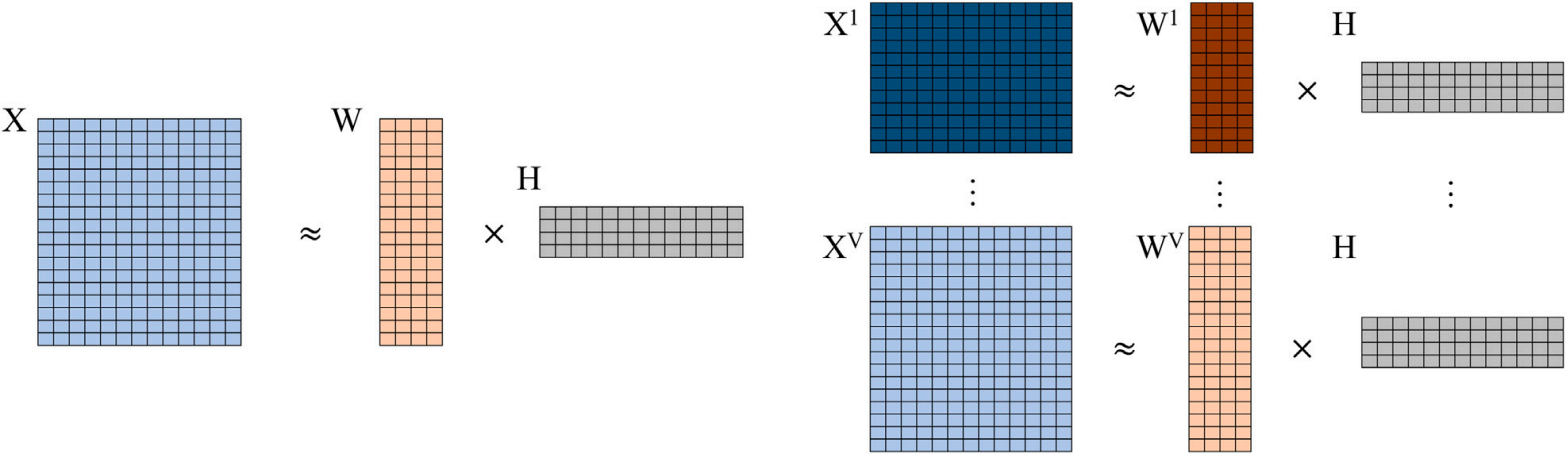
Comparison of vanilla NMF (left) to jrSiCKLSNMF without graph or sparsity constraints (right). Note that for jrSiCKLSNMF, **H** is shared among all modalities *v* ∈ 1 … *V*.

To our best knowledge, jrSiCKLSNMF is the first joint NMF method that simultaneously utilizes the KL divergence across multiple modalities of single-cell count data, a graph regularization constraint for the omics features, and a sparsity constraint for the cells. Many current methods, including Seurat, MOFA+, scAI, and CiteFuse, require using the log(*x* + 1) transformation due to the normality assumptions of these models. Similarly, using the Frobenius norm to measure the distance between two count matrices also requires the log(*x* + 1) transformation. As we mention earlier, transformation of data via the log(*x* + 1) normalization can introduce bias, especially for UMI data, because it exaggerates the difference between zero and non-zero counts and can thereby negatively impact downstream analyses (Townes et al., 2019). Since we use the Poisson KL divergence, our method does not require data to undergo this log(*x* + 1) transformation. This method extends the work done in Elyanow et al. (2020); Dai et al. (2020); Liu et al. (2020) to single-cell multimodal omics count data collected from the same set of cells. In Figure 1, we show a comparison of basic (vanilla) NMF and our developed method without sparsity constraints or graph regularization. From this parallel comparison, we can see that the **H** matrices are shared among all modalities (*v*) while the **W** ^*v*^ matrices and median library size normalized count matrices **X** ^*v*^ are different within each modality.

### 2.3 Loss functions for jrSiCKLSNMF

For our method, we concentrate on two types of loss functions: the first loss function adds a sparsity constraint on **H** and the second one sets the square root of the sum of the squared elements of the rows of **H** to sum to one. For both constraint methods, we seek to minimize the loss by using multiplicative updates (MU) (Lee and Seung, 1999). Since the constraints on **W** ^*v*^ are the same regardless of the constraints on **H**, we will describe the graph constraints and their components here. For each **W** ^*v*^, we have a graph Laplacian **L** ^*v*^ that is associated with the feature-feature similarity graph for the raw count data in modality *v*. Setting **L** ^*v*^ to the *M* ^*v*^ × *M* ^*v*^ identity matrix **I** ^*v*^, we have $\text{tr}{\left(W^{v}\right)}^{T}I^{v}W^{v}=tr\left({\left(W^{v}\right)}^{T}\left(W^{v}\right)\right)=\|W^{v}{\|}_{F}^{2}$ , which simplifies to a sparsity constraint on the square of the Frobenius norm ‖ ⋅‖_*F*_ of **W** ^*v*^. Penalty parameter $\lambda_{W^{v}}$ is a pre-specified constant for the graph regularization parameter on **W** ^*v*^ in each modality. For both loss equations, we use MU. MU is a gradient descent algorithm with an adaptive step size that ensures that all entries of every matrix at each iteration are positive. Eq. 2 defines the KL divergence between the *v* ^th^ median library size normalized omics data matrix **X** ^*v*^ and the matrix product of each reduced dimension omics feature matrix **W** ^*v*^ with shared, reduced dimension cell matrix **H**, subject to a sparsity constraint on the shared **H** and graph regularization on each **W** ^*v*^. For each **X** ^*v*^, $x_{ij}^{v}$ corresponds to the value in the *i* ^th^ row and *j* ^th^ column.

$$\begin{array}{cc} L\left(X^{v},W^{v},H\right)=\underset{W^{v},H}{\text{min}} & \overset{V}{\underset{v=1}{\sum }}\overset{M^{v}}{\underset{i=1}{\sum }}\overset{N}{\underset{j=1}{\sum }}x_{ij}^{v}\text{log}\left(\frac{x_{ij}^{v}}{{\left(W^{v}H\right)}_{ij}}\right)-x_{ij}^{v}+{\left(W^{v}H\right)}_{ij}\\ & +\frac{1}{2}\lambda_{W^{v}}\text{tr}\left({\left[W^{v}\right]}^{T}L^{v}W^{v}\right)+\frac{1}{2}\lambda_{H}\|H{\|}_{F}^{2}. \end{array}$$ (2)

Equation 3 is a similar loss functions but instead ensures that the L2 norm ‖ ⋅‖_2_ of each column of **H** equals 1.

$$\begin{array}{c} L\left(X^{v},W^{v},H\right)=\underset{W^{v},H}{\text{min}}\overset{V}{\underset{v=1}{\sum }}\overset{M^{v}}{\underset{i=1}{\sum }}\overset{N}{\underset{j=1}{\sum }}x_{ij}^{v}\text{log}\left(\frac{x_{ij}^{v}}{{\left(W^{v}H\right)}_{ij}}\right)-x_{ij}^{v}+{\left(W^{v}H\right)}_{ij}\\ +\frac{1}{2}\lambda_{W^{v}}\text{tr}\left({\left[W^{v}\right]}^{T}L^{v}W^{v}\right),\text{such that for each column }\times h\in H,\|h{\|}_{2}=1. \end{array}$$ (3)

One can also choose to use the Frobenius norm ‖ ⋅‖_*F*_ instead of the KL divergence while dealing with *V* modalities of continuous data rather than *V* modalities of count data. We thus outline the objective function with the Frobenius norm and a sparsity constraint on **H** in Eq. 4 and the objective function with column L2 norm constraints in Eq. 5:

$$L\left(X^{v},W^{v},H\right)=\underset{W^{v},H}{\text{min}}\overset{V}{\underset{v=1}{\sum }}\overset{M^{v}}{\underset{i=1}{\sum }}\overset{N}{\underset{j=1}{\sum }}\|X^{v}-W^{v}H{\|}^{2}+\frac{1}{2}\lambda_{W^{v}}\text{tr}\left({\left[W^{v}\right]}^{T}L^{v}W^{v}\right)+\frac{1}{2}\lambda_{H}\|H{\|}_{F}^{2}$$ (4)

$$\begin{array}{cc} L\left(X^{v},W^{v},H\right)=\underset{W^{v},H}{\text{min}} & \overset{V}{\underset{v=1}{\sum }}\overset{M^{v}}{\underset{i=1}{\sum }}\overset{N}{\underset{j=1}{\sum }}\|X^{v}-W^{v}H{\|}^{2}+\frac{1}{2}\lambda_{W^{v}}\text{tr}\left({\left[W^{v}\right]}^{T}L^{v}W^{v}\right),\\ & \text{such that for each column }h\in H,\|h{\|}_{2}=1. \end{array}$$ (5)

Equation 4 resembles the joint method SG-jNMF2 outlined in Dai et al. (2020); however, our method places the sparsity constraint on the shared **H** matrix and enforces graph regularization on the **W** ^*v*^ parameters in each modality while the method outlined in Dai et al. (2020) places both the graph regularization and the sparsity constraint on either the shared **H** when integrating multi-omics data or places both the graph regularization and the sparsity constraint on a shared **W** when integrating different datasets with shared features. Although we have not tested using different objective functions in different modalities (i.e., using the KL divergence in one modality and using the Frobenius norm in another), Luo et al. (2019) outline a method called Hybrid NMF (H-NMF), which identifies patient modules via a shared **H** but uses the KL divergence in the count genotypic modality and the Frobenius norm in the continuous phenotypic modality.

#### 2.3.1 Gradients of loss function

As the loss functions defined in Eqs 2, 4 do not have closed form minimizers, we apply the gradient descent optimization routine with MU proposed by Lee and Seung (1999). In contrast to traditional gradient descent, here, we compute the updates by using Hadamard (element-wise) products. Specifically, each update is equal to the element-wise product between the current value and a matrix that is the element-wise division of the negative part of the gradient by the positive part of the gradient. It is however important to note that MU updates are derived from the traditional gradient descent step, with a pre-specified rule for the step-size parameter. We compute the gradient of the loss with respect to each **W** ^*v*^ and **H** as,

$$\begin{array}{cc} \nabla_{W^{v}}L\left(X^{v},W^{v},H\right)= & \left(1_{M\times 1}\right)\left(1_{1\times N}{\left(H^{v}\right)}^{T}\right)-\left(X^{v}⊘\left(W^{v}H\right)\right){\left(H^{v}\right)}^{T}\\ & +\frac{1}{2}\lambda_{W^{v}}\left(L^{v}W^{v}+{\left(L^{v}\right)}^{T}W^{v}\right). \end{array}$$ (6)

In the case of the sparsity constraint on **H**, we provide the gradient for the loss in Eq. 7a. For the case when we enforce a unit norm constraint on the L2 norms of the rows of **H**, we also need to modify the gradient as in Eq. 7b. The procedure for calculating the gradient for this constraint is detailed for **W** in the single-modality case in Le Roux et al. (2015) and builds on work from Douglas et al. (2000) on gradient descent with unit norm constraints. This modification to the gradient avoids rescaling of **W** ^*v*^ at each iteration to ensure the unit L2 norm constraint holds for the rows of **H** and avoids saving a version of **H** that has not undergone L2 normalization.

$$\nabla_{H}L\left(X^{v},W^{v},H\right)=\overset{V}{\underset{v=1}{\sum }}{\left(W^{v}\right)}^{T}1_{M^{v}\times 1}1_{1\times N}-{\left(W^{v}\right)}^{T}\left(X^{v}⊘\left(W^{v}H\right)\right)+\lambda_{H}H,$$ (7a)

$$\begin{array}{ll} \nabla_{H}L\left(X^{v},W^{v},H\right) & =\overset{V}{\underset{v=1}{\sum }}{\left(W^{v}\right)}^{T}1_{M^{v}\times 1}1_{1\times N}-{\left(W^{v}\right)}^{T}\left(X^{v}⊘\left(W^{v}H\right)\right)\\ & \;-H⊗\left(1_{D\times D}\left({\left(W^{v}\right)}^{T}1_{M^{v}\times 1}1_{1\times N}\right)\right)+H⊗\left(1_{D\times D}\left({\left(W^{v}\right)}^{T}\left(X^{v}⊘\left(W^{v}H\right)\right)\right)\right). \end{array}$$ (7b)

We use these gradients to obtain the MU rules for each **W** ^*v*^ and for **H**.

### 2.4 Computation

Fitting NMF models to omics data entails many challenges, including appropriate data pre-processing, normalization, and algorithmic initialization of NMF. For clarity, we explain these steps in Sections 2.4.1–2.4.4 before providing an overview of the algorithm in Section 2.4.5.

#### 2.4.1 Quality control and normalization

Before applying the algorithm, we must perform quality control (QC) and normalization. These are vital steps for downstream analyses (Ellis et al., 2021). For QC, it is appropriate to perform standard QC, including filtering out low-quality cells, such as those with a high percentage of mitochondrial genes, low gene expression, or very high gene expression in the scRNA-seq modality. For both of the datasets we used in our analysis, this QC step was already performed. Since we develop this method primarily for multimodal single-cell data, from now on, we refer to “observations” as “cells” and “features” as “omics features.” In the case of scRNA-seq data, the entries of the data before median library size normalization would be the UMI counts; and for scATAC-seq data, these are the counts of accessible peaks/bins. To generate the median library size normalized matrix **X** ^*v*^ for each modality *v*, we first divide the counts in each cell by the sum of counts within that cell (i.e., the library size) and then multiply all entries by the median library size (Zheng et al., 2017; Elyanow et al., 2020). This does not violate count assumptions for the Poisson distribution. We use the KL divergence to measure the discrepancy between the distributions Poisson(**X** ^*v*^) and Poisson(**W** ^*v*^ **H**).

#### 2.4.2 Construction of the **L** ^*v*^ matrices

The **L** ^*v*^ matrix is the *M* ^*v*^ × *M* ^*v*^ graph Laplacian matrix of **G** ^*v*^. **G** ^*v*^ is an *M* ^*v*^ × *M* ^*v*^ interaction network graph within the *v* ^th^ omics modality. We construct **L** ^*v*^ from the raw data rather than from the median library size normalized data. To construct the graph Laplacian matrix **L** ^*v*^, one first needs to define **A** ^*v*^, the adjacency matrix of **G** ^*v*^, and **D** ^*v*^, the diagonal matrix of vertex degrees of **G** ^*v*^. The graph Laplacian matrix is defined as **L** ^*v*^ = **D** ^*v*^ − **A** ^*v*^ (Merris, 1994). Optimally, to construct **G** ^*v*^, one would use data from a different single-cell experiment on the same tissue or from a bulk experiment on the same tissue to avoid overfitting. **G** ^*v*^ is not restricted to a specific kind of graph; this method can accommodate the use of any graph that accurately captures the similarity between features (Cai et al., 2008; 2011). The use of co-expression networks from bulk tissue studies is also permissible (Elyanow et al., 2020). In our analyses, we use k-nearest neighbor (KNN) graphs as implemented in the scran package (Lun et al., 2016) to generate the graph **G** ^*v*^ for each modality. We also tested using shared nearest neighbor (SNN) graphs; however, regularization using KNN outperformed these SNN graphs. Because we are calculating the feature-feature similarities and *M* ^*v*^ ≫ *N* for all modalities *v*, distances calculated in Euclidean space for the KNN graph are meaningful. In the case when *N* > *M* ^*v*^, we would need a different approach for constructing graphs. Since we perform this graph construction on feature-feature networks, we will, without loss of generality, refer to each point within the constructed graph as a feature.

#### 2.4.3 Determination of *D* and initialization of the **W** ^*v*^ matrices and the **H** matrix

An important aspect of using any NMF-based method to analyze data matrix **X** ^*v*^ is the determination of the number of latent factors *D* and the initialization of matrices **W** ^*v*^ and **H**. Since our method of identifying an appropriate number of latent factors requires initializing and updating **W** ^*v*^ and **H**, we will discuss their initialization first. Random initialization is a common way to initialize **W** and **H** (Lee and Seung, 1999; Cai et al., 2008; Elyanow et al., 2020; Liu et al., 2020), but many other methods of initialization have been developed over the years. In particular, initialization based on singular-value decomposition (SVD) has become increasingly popular (Boutsidis and Gallopoulos, 2008; Qiao, 2015; Esposito, 2021) as a way of initializing non-negative matrix factorization problems. To initialize **W** ^*v*^ for each modality, we first perform Non-negative Double Singular Value Decomposition (NNDSVD), a method developed by Boutsidis and Gallopoulos (2008) for NMF initialization, on each **X** ^*v*^ and use the **W** ^*v*^ matrices from each output. To initialize **H**, we concatenate all **X** ^*v*^ together to generate **X** ^*all*^, perform NNDSVD on this concatenated matrix, and then use the **H** matrix from the NNDSVD output. While NNDSVD encourages a sparse initialization, because we use MU which cannot escape from 0 values, we use a dense initialization where we insert the average value instead of 0. Additionally, since NNDSVD is a non-negative version of singular value decomposition, the sum of each eigenvector decreases for each component as the number of factors increases. This is not necessarily the case for NMF. We therefore initialize **W** ^*v*^ such that each column sums to the mean column sum. We perform this same operation on the rows of **H**. We also tested using random initialization, which, due to ease of implementation, is a common method of initialization. It did not perform as accurately as NNDSVD and, on simulated data with no added noise, an individual regularization graph, and a sparsity constraint on **H**, had an adjusted Rand index (ARI) (Hubert and Arabie, 1985) of 0.886, which was about 10% lower than the 0.988 achieved using NNDSVD. We provide side-by-side boxplots of these results on simulated data in Supplementary Figure S1.

It can be difficult to identify an appropriate *D* for unsupervised data problems like clustering. In our workflow, we provide a method of visual selection. We initialize the **W** ^*v*^ and **H** matrices for a user-specified range of number of factors (default is 2–20) under either NNDSVD or random initialization (we strongly recommend NNDSVD). We then run the algorithm for a specified number of iterations (100 for sparsity constraint and 1 for the L2 Norm constraint) and then plot the resulting loss function. We recommend selecting the number of latent factors that corresponds to the elbow of the plot. We provide an example of this on real data in Section 3 in Figure 6. The computational time increases with increasing *D*; for an example of this, see Supplementary Figure S2.

#### 2.4.4 Selection of *λ* values

Selection of the *λ* values is a time-intensive step. As the number of modalities increases, the selection step becomes even more time demanding. We thus run extensive simulations for scRNA-seq and scATAC-seq data and, using these simulations, identify some default choices for these parameters. Based on our experiments we find that $\lambda_{W^{\mathrm{RNA}}}=10$ , $\lambda_{W^{\mathrm{ATAC}}}=50$ , and *λ*_*H*_ = 500 work well for the sparsity constraint model and that $\lambda_{W^{\mathrm{RNA}}}=3$ , $\lambda_{W^{\mathrm{ATAC}}}=15$ work well for the L2 norm constraint on the rows of **H**. To illustrate this, we provide a plot of the ARI values for 512 combinations of $\lambda_{W^{\mathrm{RNA}}}$ , $\lambda_{W^{\mathrm{ATAC}}}$ , and *λ*_*H*_ in Supplementary Figure S3 for a fixed *D* = 10 for the no-added-noise simulated data.

We recommend $\lambda_{W^{\mathrm{RNA}}}=10$ , $\lambda_{W^{\mathrm{ATAC}}}=50$ , and *λ*_*H*_ = 500 as the default for the sparsity constraint model and $\lambda_{W^{\mathrm{RNA}}}=3$ , $\lambda_{W^{\mathrm{ATAC}}}=15$ for the model with the L2 norm constraint on the rows of **H** as the default choices for all of our simulations and our real data application. The value of 10 for the RNA modality agrees with previous literature for KL-based NMF (KL-NMF) algorithms on scRNA-seq data (Elyanow et al., 2020). Finally, the computational time does not seem highly dependent on these values, but we do see faster computational times for $\lambda_{W^{\mathrm{RNA}}}=\lambda_{W^{\mathrm{ATAC}}}=1000$ . We plot these in Supplementary Figure S4. However, $\lambda_{W^{\mathrm{RNA}}}=\lambda_{W^{\mathrm{ATAC}}}=1000$ are not considered due to their poor performance.

#### 2.4.5 Overview of algorithm

**FIGURE 2:**
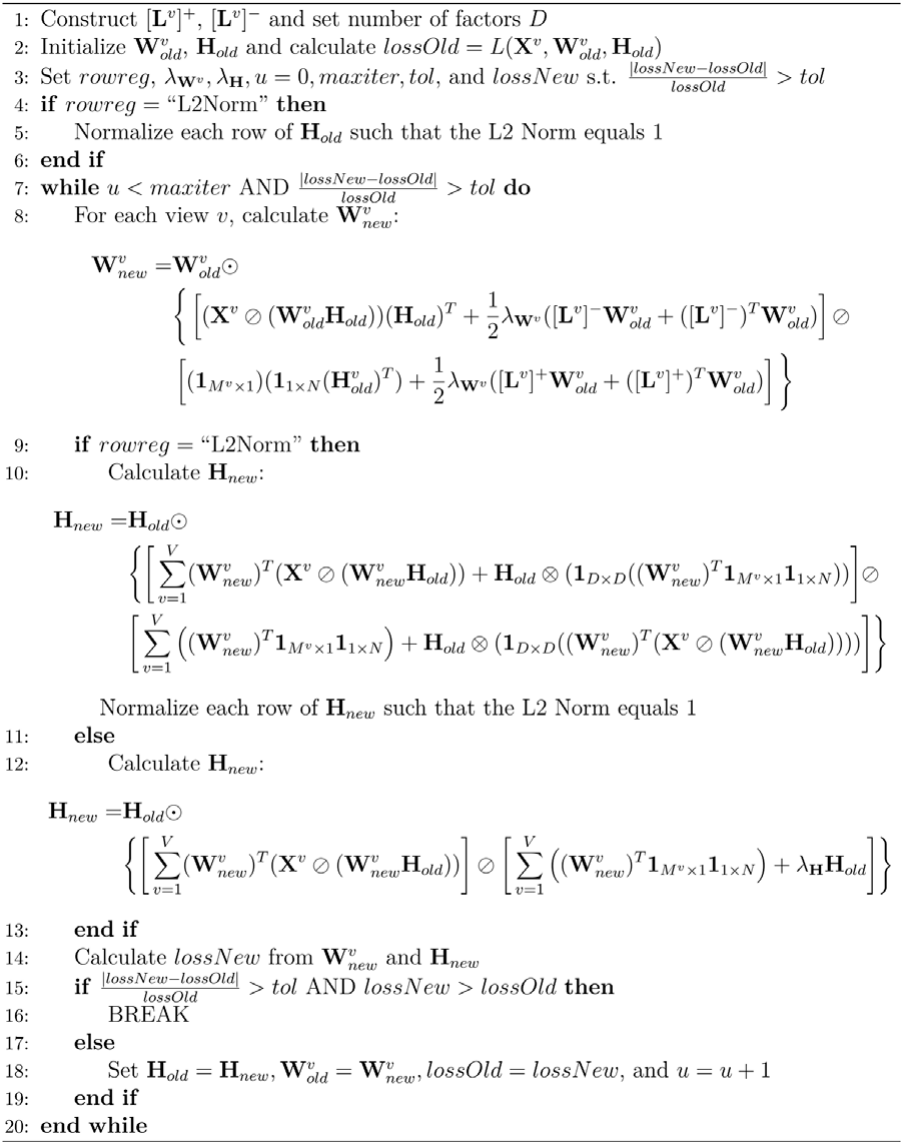
Pseudocode for jrSiCKLSNMF. Note that the sparsity parameter is not included in the row regularization. While it is possible to use both the *λ*_**H**_ sparsity parameter and the unit L2 norm constraint on the rows of **H**, it is not necessary. Since we did not use both constraints simultaneously, to save space, we are excluding the *λ*_**H**_ term when *rowreg* = “L2Norm” from the calculation of **H**_*u*+1_.

The pseudocode in Figure 2 summarizes all the steps for the jrSiCKLSNMF algorithm. First, we must construct the graph-Laplacian matrices from feature-feature similarity graphs and select a number of factors *D* that we wish to use to construct the **W** ^*v*^ matrices and the **H** matrix. Note that *D* must be the same across all modalities. Next, we set the $\lambda_{W^{v}}$ values, the *λ*_**H**_ value, the update tolerance, and the new loss. The *λ* values are tuning parameters. For our simulations, we set the maximum number of iterations to 10,000 and the tolerance to 10^–6^ for both the sparsity and the L2 norm constraint. Then, using MU we iteratively update **W** ^*v*^ and **H** until convergence (i.e., the percentage difference of the update is less than the tolerance) or we reach a maximum number of iterations. In line 8 of Figure 2 we show the multiplicative updates for $W_{u+1}^{v}$ , the (*u* + 1)^th^ updates of the **W** ^*v*^ matrices in sequence, using the corresponding feature matrices $W_{u}^{v}$ and cell matrix **H**_*u*_. Similarly, in line 10 of Figure 2, we show the calculations:

$$\begin{array}{cc} W_{u+1}^{v}= & W_{u}^{v}⊙\left\{\left[\left(X^{v}⊘\left(W_{u}^{v}H_{u}\right)\right){\left(H_{u}\right)}^{T}+\frac{1}{2}\lambda_{W^{v}}\left({\left[L^{v}\right]}^{-}W_{u}^{v}+{\left({\left[L^{v}\right]}^{-}\right)}^{T}W_{u}^{v}\right)\right]⊘\right.\\ & \left.\left[\left(1_{M\times 1}\right)\left(1_{1\times N}{\left(H_{u}\right)}^{T}\right)+\frac{1}{2}\lambda_{W^{v}}\left({\left[L^{v}\right]}^{+}W_{u}^{v}+{\left({\left[L^{v}\right]}^{+}\right)}^{T}W_{u}^{v}\right)\right]\right\}. \end{array}$$ (8)

here, ${\left[L^{v}\right]}^{-}$ and ${\left[L^{v}\right]}^{+}$ indicate the absolute values of the negative and the positive parts of the **L** ^*v*^ in each modality, respectively, the ⊙ symbol indicates the Hadamard product, and the ⊘ symbol indicates Hadamard division. After updating all **W** ^*v*^ matrices, we proceed to updating **H**_*u*+1_ from the new $W_{u+1}^{v}$ matrices and the old **H**_*u*_ matrix via Eq. 9a for the sparsity constraint on **H** and Eq. 9b for the L2 norm constraint.

$$H_{u+1}=H_{u}⊙\left\{\left[\overset{V}{\underset{v=1}{\sum }}{\left(W_{u+1}^{v}\right)}^{T}\left(X^{v}⊘\left(W_{u+1}^{v}H_{u}\right)\right)\right]⊘\left[\overset{V}{\underset{v=1}{\sum }}\left({\left(W_{u+1}^{v}\right)}^{T}1_{M\times 1}\left(1_{1\times N}\right)\right)+\lambda_{H}H_{u}\right]\right\},$$ (9a)

$$\begin{array}{ll} H_{u+1} & =H_{u}⊙\\ & \left\{\left[\overset{V}{\underset{v=1}{\sum }}{\left(W_{u+1}^{v}\right)}^{T}\left(X^{v}⊘\left(W_{u+1}^{v}H_{u}\right)\right)+H_{u}⊗\left(1_{D\times D}\left({\left(W_{u+1}^{v}\right)}^{T}1_{M^{v}\times 1}1_{1\times N}\right)\right)\right]\right.\\ & ⊘\left.\left[\overset{V}{\underset{v=1}{\sum }}\left({\left(W_{u+1}^{v}\right)}^{T}1_{M^{v}\times 1}1_{1\times N}\right)+H_{u}⊗\left(1_{D\times D}\left({\left(W_{u+1}^{v}\right)}^{T}\left(X^{v}⊘\left(W_{u+1}^{v}H_{u}\right)\right)\right)\right)\right]\right\}. \end{array}$$ (9b)

This process of iterative updates continues until the algorithm converges.

### 2.5 Clustering

In our *post hoc* analysis of the simulated data, we perform k-means clustering on the estimated **H** matrix. For fair comparison, we set the number of clusters to be equal to the true value. For Seurat, which uses a resolution parameter rather than the number of clusters, we experiment with a subset of the data to determine a suitable resolution parameter that ensures that the number of clusters is close to 3. One may use any clustering method to perform clustering on the consensus matrix **H**, including using the **H** matrix itself as a clustering algorithm. We use k-means because we can set the number of clusters to the true number of clusters easily, and it has good clustering performance. To aid in the determination of the number of clusters on real datasets, we provide wrapper functions for the R packages nbClust (Charrad et al., 2014) and clValid (Brock et al., 2008). These packages generate validation metrics and plots to help in determining the ideal number of clusters for k-means and other clustering methods.

## 3 Results

To compare the performance of our algorithm against other methods, we perform a simulation study. Since our algorithm is for use with exploratory data analyses and clustering, it is somewhat difficult to evaluate its performance on a real dataset where the true clusters are unknown. We use GSE130399 (Zhu et al., 2019), which is labeled, to generate parameters from which to simulate datasets, and GSE126074 (Chen et al., 2019), which has an annotation but is not labeled, to assess the performance of our algorithm on a real data example. To perform our simulation study, we use two different R packages: SPARSim (Baruzzo et al., 2020) for scRNA-seq data simulation and simATAC (Navidi et al., 2021), for scATAC-seq data simulation. We generate all plots using the R package ggplot2 version 3.4.2 (Wickham, 2016).

### 3.1 Evaluation metrics for clustering

To determine the accuracy of clusters and compare these clusters to other methods, we use the ARI as implemented in the R package aricode. We use this to evaluate how the clusters identified by each method compare to the ground truth in the simulated data and to the annotations for the real data. The ARI uses the hypergeometric distribution to correct for clusters that are correct due to random chance. We also explored comparison of the adjusted mutual information (AMI) (Xuan Vinh et al., 2009), and the results were similar.

### 3.2 Simulation study

For the simulation study, we simulate four sets of 100 independent dual-assay scRNA-seq/scATAC-seq datasets, each with increasing amounts of added noise starting from 0. Each dataset consists of 100 cells each of 3 different cell types for a total of 300 cells. There are approximately 900 genes in the scRNA-seq modality and approximately 5,800 bins in the scATAC-seq modality for the simulated cells. These vary marginally among simulations. In the next section, we discuss the reasoning behind this number of genes and bins. We use this labeled simulated data to determine *λ* values as well by examining different combinations of *λ* values and their corresponding ARIs. We then choose the values that correspond to the highest average ARI.

#### 3.2.1 Data simulation scheme

To simulate the data jointly, we use the R packages SPARSim (Baruzzo et al., 2020) to simulate scRNA-seq expresion and simATAC (Navidi et al., 2021) to simulate scATAC-seq expression. We estimate simulation parameters from GSE130399, a real Paired-seq (Zhu et al., 2019) cell-line dataset. SPARSim estimates parameters from real data and then uses a Gamma-Multivariate hypergeometric mixture model to simulate scRNA-seq count data. simATAC also estimates parameters from real data but uses a Bernoulli-Poisson hurdle model to generate data. To prepare the data for parameter estimation, we perform aggressive quality control using the R packages Seurat (Satija et al., 2015) and Signac (Stuart et al., 2021) for the scRNA-seq modality and scATAC-seq modality, respectively. First, we exclude cells which have fewer than 400 and greater than 2000 RNA counts and cells that have fewer than 300 or greater than 4000 ATAC bins. In the RNA modality, we exclude genes with fewer than 10 counts per cell and in the ATAC modality, we exclude bins with fewer than 20 counts per cell as in Zhu et al. (2019).

We then select the 1,000 most highly variable genes in the RNA-seq modality and the features that are common among 95% of the cells in the ATAC-seq modality. After performing this quality control and feature selection, we are left with 382 HEK293 cells, 366 HepG2 cells, and 1,003 mix cells from which to sample. To generate each of the 100 datasets, we randomly select 100 HepG2 cells, 100 HEK293 cells, and 100 mix cells without replacement. The mix cells are a mixture of the HepG2 and HEK293T cells; however, for the purpose of generating data for this simulation, we treat them as a third cell type. From this subset, we then use SPARSim to estimate simulation parameters and finally generate cells for the RNA modality and use simATAC to estimate simulation parameters and generate simulated cells for the ATAC modality. To avoid confusion between modalities, instead of using *M* ^*v*^, we use *M* ^*RNA*^ to denote the number of features in the scRNA-seq modality and *M* ^*ATAC*^ to denote the number of features in the scATAC-seq modality. As mentioned earlier, we generate four sets of datasets; one with no noise and three with increasing amounts of noise. SPARSim and simATAC simulate added noise differently; SPARSim uses an estimated variability parameter and simATAC adds noise from a Gaussian distribution to the final dataset. Therefore, in our simulation study, we follow the respective protocols for adding noise to each modality. For the lowest added noise datasets, for each simulated dataset, we generate noise from a uniform distribution (U(1,1.25)) and multiply this noise by the corresponding variability parameter for each RNA feature. In the ATAC modality, we simulate the data in simATAC and then, following the protocol for generating noise, add Gaussian noise generated from normal distribution ( N(−0.25,0.25) ) for each entry in the **X** ^*ATAC*^ matrix. We repeat this noise generation process twice more, using distributions [ U(1,1.5) , N(−0.5,0.5) ] and [ U(1,2) , N(−1,1) ].

### 3.3 Current single-cell multimodal omics methods

Since this is a relatively new technology, we compare our method to five other methods of integrating single-cell multimodal omics data. These methods are not necessarily designed for use with dual scRNA-seq and scATAC-seq data. These methods are BREM-SC (Wang et al., 2020), Seurat v. 4.0, MOFA+, scAI, and CiteFuse. We briefly describe them in Table 1 and describe them in more detail in sub-subSections 3.3.1–3.3.4. This is not an exhaustive list of methods, and all of these methods are implemented in R. There are other methods that are implemented in Python (Van Rossum and Drake, 2009) that we do not discuss here. Each of these methods can work with, at a minimum, two modalities of simultaneous measurements of omics data on the same set of single cells. Some, like MOFA+, can work with more than two modalities. While the focus of our comparisons is on these 5, there are other methods of integrating data across omics profiles.

**TABLE 1:** Methods for comparison to jrSiCKLSNMF with a brief description *Seurat has also been successfully used on dual assay scRNA-seq/scATAC-seq but was developed for CITE-seq data.

Method	Data designed for	Type of model
BREM-SC	CITE-seq	Bayesian random effects mixture
CiteFuse	CITE-seq	Similarity Network Fusion
MOFA+	Any two -omics datasets	Factor Analysis
scAI	scRNA-seq and ATAC-seq	Non-negative Matrix Factorization
Seurat v. 4	CITE-seq*	Weighted Nearest Neighbor

#### 3.3.1 BREM-SC

The Bayesian random effects mixture model for single-cell multi-omics data (BREM-SC) model is intended for use on data collected from cellular indexing of transcriptomes and epitopes by sequencing (CITE-seq). These are joint RNA and Antibody-Derived Tags (ADT) single-cell data. ADT data are much lower dimension than scRNA-seq since it only works with a few proteins per cell; in Stoeckius et al. (2017), which introduces CITE-seq, the number of features in the ADT modality is 13 (Stoeckius et al., 2017). BREM-SC uses a Bayesian Dirichlet-multinomial model with cell-specific random effects shared between the two modalities to perform cell clustering. In Eq. 10, we provide the complete log likelihood for BREM-SC:

$$\begin{array}{cc} & \log P\left(\right.\left.\alpha^{\mathrm{RNA}},\alpha^{\mathrm{ADT}},Z,b_{j}|X^{\mathrm{RNA}},X^{\mathrm{ADT}}\right)\propto \overset{C}{\underset{j=1}{\sum }}\overset{K}{\underset{k=1}{\sum }}I\left(z_{j}=k\right)\\ & \times \log\left\{\left(\overset{G}{\underset{i=1}{\prod }}\frac{\Gamma \left(x_{ij}^{\mathrm{RNA}}+\alpha_{i\left(k\right)}^{\mathrm{RNA}}b_{j}\right)}{\Gamma \left(\alpha_{i\left(k\right)}^{\mathrm{RNA}}b_{j}\right)}\right)\frac{\Gamma \left(\left|\alpha_{\left(k\right)}^{\mathrm{RNA}}b_{j}\right|\right)}{\Gamma \left(T_{j}^{\mathrm{RNA}}+\left|\alpha_{\left(k\right)}^{\mathrm{RNA}}b_{j}\right|\right)}\right.\\ & \times \left(\overset{D}{\underset{d=1}{\prod }}\frac{\Gamma \left(x_{dj}^{\mathrm{ADT}}+\alpha_{d\left(k\right)}^{\mathrm{ADT}}b_{j}\right)}{\Gamma \left(\alpha_{d\left(k\right)}^{\mathrm{ADT}}b_{j}\right)}\right)\times \left.\frac{\Gamma \left(\left|\alpha_{\left(k\right)}^{\mathrm{ADT}}b_{j}\right|\right)}{\Gamma \left(T_{j}^{\mathrm{ADT}}+\left|\alpha_{\left(k\right)}^{\mathrm{ADT}}b_{j}\right|\right)}\right\}+\overset{C}{\underset{j=1}{\sum }}\left(-\log b_{j}-\frac{{\left(\log b_{j}\right)}^{2}}{2\sigma_{b}^{2}}\right)\\ & \overset{C}{\underset{j=1}{+\sum }}\left(-\frac{1}{2}\log\sigma_{b}^{2}\right) \end{array}$$ (10)

***α*** ^*RNA*^, a *G* gene by *K* cluster matrix and ***α*** ^*ADT*^, a *D* protein marker by *K* matrix, contain the cell cluster-specific Dirichlet parameters for the RNA and ADT modalities, respectively. $\alpha_{i\left(k\right)}^{\mathrm{RNA}}$ is the value for gene *i* in cluster *k* of ***α*** ^*RNA*^, and $\alpha_{d\left(k\right)}^{\mathrm{ADT}}$ is the value for protein marker *d* in cluster *k* of ***α*** ^*ADT*^. $\alpha_{\left(k\right)}^{\mathrm{RNA}}$ and $\alpha_{\left(k\right)}^{\mathrm{ADT}}$ are the vectors of Dirichlet priors for the *k* ^th^ cell cluster in the RNA and ADT modalities, respectively. If cell *j* belongs to the *k* ^th^ cell type, its gene expression profile $p_{j}^{\mathrm{RNA}}$ follows cell-type-specific prior distribution $\text{Dir}\left(\alpha_{\left(k\right)}^{\mathrm{RNA}}\right)$ , and its marker expression profile $p_{j}^{\mathrm{ADT}}$ follows $\text{Dir}\left(\alpha_{\left(k\right)}^{\mathrm{ADT}}\right)$ . *Z* is a latent variable vector comprised of elements *z*_*j*_ that represent the cell type label *k* ∈ (1, … *K*) for each cell *j* ∈ (1, … , *C*). Here, *C* is the total number of cells, and *K* is the total number of cell labels. *b*_*j*_ is the random effect for the *j* ^th^ cell and follows distribution $\text{LogNormal}\left(0,\sigma_{b}^{2}\right)$ , where $\sigma_{b}^{2}$ indicates the among-cell variability. **X** ^*RNA*^ and **X** ^*ADT*^ are the *G* gene by *C* RNA data matrix and *D* protein marker by *C* ADT data matrix, respectively. *I*(⋅) is the indicator function and Γ(⋅) is the gamma function. $x_{ij}^{\mathrm{RNA}}$ is the entry in the *i* ^th^ row and *j* ^th^ column of **X** ^*RNA*^ while $x_{dj}^{\mathrm{ADT}}$ is the entry in the *d* ^th^ row and *j* ^th^ column of **X** ^*ADT*^. Finally, $T_{j}^{\mathrm{RNA}}$ and $T_{j}^{\mathrm{ADT}}$ are the total UMI counts and the total ADT counts, respectively for the *j* ^th^ cell. BREM-SC uses a Gibbs sampler to update cluster assignment *z*_*j*_ and uses a random walk Metropolis within Gibbs sampler to iteratively update $\alpha_{\left(k\right)}^{\mathrm{RNA}}$ , $\alpha_{\left(k\right)}^{\mathrm{ADT}}$ , and *b*_*j*_.

#### 3.3.2 CiteFuse

CiteFuse, like BREM-SC, is also intended for CITE-Seq (dual assay scRNA-seq and single-cell ADT) data. It uses similarity network fusion to integrate the two modalities. First, CiteFuse performs a centered log-ratio transformation to normalize the ADT counts. Next, it calculates cell-to-cell similarity by using a similarity metric called *perb* from R package propr (Quinn et al., 2017). For the RNA expression, it uses Pearson’s correlation on highly variable genes identified by scran. It then scales the matrices using an exponential similarity kernel and fuses them via a similarity network fusion algorithm (Wang et al., 2014). To compare to our method, we use *perb* for the scRNA-seq data and Pearson’s correlation for scATAC-seq because, as scRNA-seq data are sparser and noisier than ADT data, so too are scATAC-seq data sparser and noisier than scRNA-seq data.

#### 3.3.3 MOFA+

Multi-omics Factor Analysis v2 (MOFA+) captures global sources of variability in multi-omics data in a small number of latent factors via a Bayesian matrix factorization framework. MOFA+ can be used on single-cell data, grouped data, and is available for more than two modalities. Eq. 11 gives the underlying equation for the matrix factorization model:

$$Y_{gm}=Z_{g}W_{m}^{T}+\epsilon_{gm}.$$ (11)

Here, **Y**_*gm*_ is a matrix of observations of the *m* ^th^ modality and *g* ^th^ group. For single-cell data, group indicates the source of the tissue. **W**_*m*_ is a weight matrix for the *m* ^th^ modality, **Z**_*g*_ is the factor matrix for the *g* ^th^ group and ***ϵ***_*gm*_ represents the residual for the *m* ^th^ modality and the *g* ^th^ group. Each **Z**_*g*_ is of dimension *N*_*g*_ × *K*, where *N*_*g*_ is the number of observations per group and *K* is the number of latent factors. $W_{m}^{T}$ has dimension *D*_*m*_ × *K*, where *D*_*m*_ is the number of features in the *M* ^th^ modality. It also uses regularization for both the factors and weights in the form of an Automatic Relevance Determination (ARD) prior to model activity of factors across modalities or sample groups and a spike-and-slab prior to encourage sparsity.

#### 3.3.4 scAI

scAI, like our method, is based on NMF. Eq. 12 is the loss function for scAI where *M* ^1^ genes by *N* cells matrix **X**_1_ and *M* ^2^ loci by *N* cells matrix **X**_2_ correspond to RNA and ATAC modalities, respectively. **W**_1_ is an *M* ^1^ by *D* factors gene loading matrix, **W**_2_ is an *M* ^2^ by *D* loci loading matrix, **H** is the *D* × *N* cell loading matrix where **H**_. *j*_ is the *j* ^th^ column of **H**, the **Z** matrix is a cell-cell similarity matrix, ◦ represents dot multiplication, **R** is a binary matrix generated by a binomial distribution with probability *s*, and *α*, *λ*, and *γ* are regularization parameters. Like our method, it shares the **H** matrix but, unlike our method, it binarizes the ATAC-seq modality of the data.

$$\underset{W_{1},W_{2},H,Z\geq 0}{\min}\alpha {\left\|X_{1}-W_{1}H\right\|}_{F}^{2}+{\left\|X_{2}\left(Z◦R\right)-W_{2}H\right\|}_{F}^{2}+\lambda {\left\|Z-H^{T}H\right\|}_{F}^{2}+\gamma \underset{j}{\sum }{\left\|H_{.j}\right\|}_{1}^{2}$$ (12)

Interestingly, even though this algorithm is fairly similar to ours, their implementation performs poorly in our comparative study on simulated data. This may illustrate the importance of the graph regularization constraints.

#### 3.3.5 Seurat

Seurat v.4 uses weighted nearest neighbor (WNN) to integrate bimodal single-cell data. Like BREM-SC and CiteFuse, it was developed for CITE-seq data; however it has also been used for scATAC-seq and scRNA-seq dual assay data. After quality control, normalization, and dimension reduction on each modality, Seurat constructs independent KNN graphs for both modalities. Next, it performs within and across-modality prediction and cell-specific modality weights:

$$\theta_{\text{weighted }}\left(i,j\right)=w_{\text{RNA }}\left(i\right)\theta_{\text{RNA}}\left(r_{i},r_{j}\right)+w_{\text{protcin }}\left(i\right)\theta_{\text{protein }}\left(p_{i},p_{j}\right).$$ (13)

*θ*_weighted_ (*i*, *j*) is the weighted similarity between cells *i* and *j*, *w*_rna_ (*i*) is the cell-specific RNA weight, $\theta_{\text{RNA}}\left(r_{i},r_{j}\right)$ is the affinity between the RNA profiles of cells *i* and *j*, *w*_protein_ (*i*) is the cell-specific ADT weight, $\theta_{\text{protein}}\left(r_{i},r_{j}\right)$ is the affinity between the ADT profiles of cells *i* and *j*. Then, a final KNN graph is constructed using *θ*_weighted_ (*i*, *j*) as the similarity metric. To identify clusters, Seurat uses community detection algorithms on this graph.

### 3.4 Comparison to other methods on simulated data

**FIGURE 3:**
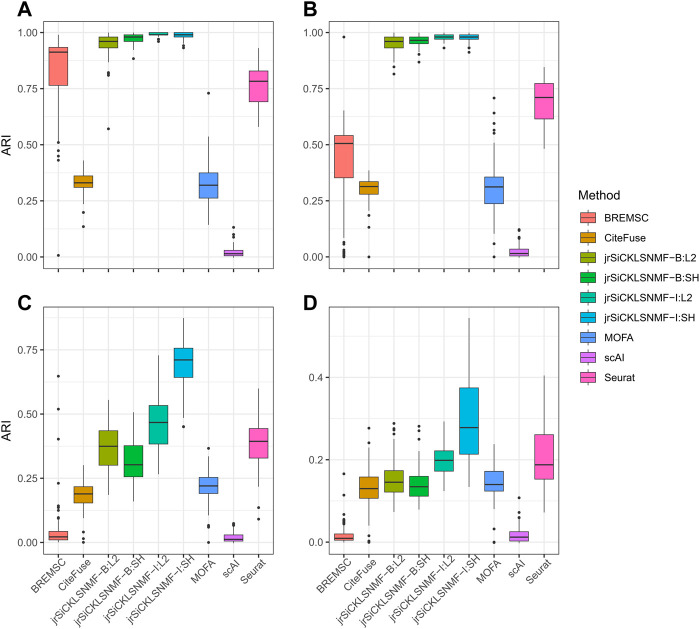
Comparison of different versions of jrSiCKLSNMF to other methods. A “B” in the method indicates that the regularizing graph is generated from bulk data while an “I” indicates that the regularizing graph is generated from the data itself. “SH” indicates that a sparsity parameter is included on **H** while “L2” indicates that the L2 norms of the rows of **H** are equal to 1. For all simulations, we generate 10 latent NMF factors. For all “SH,” *λ* **W** ^*RNA*^ = 10, *λ* **W** ^*ATAC*^ = 50, *λ*_*H*_ = 500. For all “L2,” *λ* **W** ^*RNA*^ = 3, *λ* **W** ^*ATAC*^ = 15. **(A)** Data simulated for the RNA and ATAC modalities from SPARSim and SimATAC, respectively, with no added noise. **(B)** The gene variability parameter is increased by up to 25% in the RNA simulation and noise simulated from N(−0.25,0.25) distribution is added to the ATAC simulation. **(C)** The gene variability parameter is increased by up to 50% in the RNA simulation and noise simulated from N(−0.5,0.5) distribution is added to the ATAC simulation. **(D)** The gene variability parameter is increased by up to 100% in the RNA simulation and noise simulated from N(−1,1) distribution is added to the ATAC simulation. Note that here, BREMSC is unable to run on dataset 99.

We compare our method to the five methods discussed in the previous section. The numerical comparisons are illustrated in Table 2, with the best performing value in bold. For every level of noise, a version of jrSiCKLSNMF performed best in terms of ARI. We include four variants of jrSiCKLSNMF in our comparison: jrSiCKLSNMF-B:L2, jrSiCKLSNMF-B:SH, jrSiCKLSNMF-I:L2, and jrSiCKLSNMF-I:SH. The first variant, jrSiCKLSNMF-B:L2, is jrSiCKLSNMF with graph regularization term **L** ^*v*^ constructed from a feature-feature KNN graph built from simulated bulk data (i.e., **L** ^*v*^ is the same for all 400 datasets). jrSiCKLSNMF-B:L2 also has a unit L2 norm constraint on the rows of **H**. For the second variant jrSiCKLSNMF-B:SH, the **L** ^*v*^ used is the same as the one used in jrSiCKLSNMF-B:L2, but there is a sparsity constraint on **H**. For the third variant jrSiCKLSNMF-I:L2, **L** ^*v*^ is different for each of the 400 datasets and is constructed individually from each dataset’s feature-feature KNN graph. It also, like jrSiCKLSNMF-B:L2, has a unit L2 norm constraint on the rows of **H**. The final variation jrSiCKLSNMF-I:SH has individual **L** ^*v*^ matrices for each dataset as in jrSiCKLSNMF-I:L2 and has a sparsity constraint on **H** as in jrSiCKLSNMF-B:SH. Except for MOFA+, which was run on a 16.0 RAM local machine due to difficulty with setting up Python modules from reticulate (Ushey et al., 2023) on the cluster, we ran all analyses on the HiPerGator 3.0 high performance cluster. As such, MOFA + may have slightly faster mean running times listed here than it would if it were run on the cluster. For the jrSiCKLSNMF analyses, we used 3 GB of RAM per node, and for the other methods, we used 10 GB of RAM on the high performance cluster for most analyses. BREM-SC sometimes had high RAM requirements and failed to run on all datasets, so we tested using up to 50 GB when needed. BREM-SC also required the manual re-setting of the random seed when it failed to converge for certain datasets. In addition to Table 2, in Figure 3, we also provide boxplots of results from our method along with results from other R-based methods. Not only is our method more accurate, it also has a very low variability, indicating that it works similarly over many different datasets. We can also see from this that our method is robust to increased noise; jrSiCKLSNMF, with graph Laplacian **L** ^*v*^ constructed from each individual dataset’s feature-feature similarity and a sparsity constraint on **H**, consistently outperforms other methods for all noise levels.

**TABLE 2:** Here, we provide the mean ARI, median ARI, standard deviation of ARI, and the mean running time for BREM-SC, Citefuse, jrSiCKLSNMF-B:L2, jrSiCKLSNMF-B:SH, jrSiCKLSNMF-I:L2,jrSiCKLSNMF-I:SH, MOFA+, scAI, and Seurat on 400 simulated datasets (100 datasets in each of 4 noise conditions). Bold entries indicate the best performance in each column. Note that these times include all normalization and pre-processing steps required to run each algorithm. We use the Seurat normalization workflow to normalize the data for MOFA+, so Seurat normalization is included as part of its computation time. A variant of jrSiCKLSNMF performs best for all examples, and CiteFuse, when compared using all pre-processing steps, has the fastest performance. Bold values indicate the best performing algorithm per column.

No added noise	Mean	Median	Standard deviation	Mean time
BREM-SC	0.827	0.913	0.178	615.75 s
CiteFuse	0.330	0.330	0.0449	**6.87 s**
jrSiCKLSNMF-B:L2	0.949	0.960	0.0540	84.46 s
jrSiCKLSNMF-B:SH	0.974	0.980	0.0199	65.82 s
jrSiCKLSNMF-I:L2	**0.992**	**0.990**	0.0095	60.04 s
jrSiCKLSNMF-I:SH	0.988	**0.990**	0.0127	46.77 s
MOFA+	0.321	0.320	0.0971	11.39 s
scAI	0.021	0.014	0.0240	149.25 s
Seurat	0.767	0.783	0.0857	31.01 s
U(1,1.25),N(−0.25,0.25)	Mean	Median	Standard Deviation	Mean Time
BREM-SC	0.427	0.505	0.179	1,181.96 s
CiteFuse	0.303	0.314	0.0540	**8.89 s**
jrSiCKLSNMF-B:L2	0.952	0.960	0.0327	84.93 s
jrSiCKLSNMF-B:SH	0.961	0.965	0.0223	58.43 s
jrSiCKLSNMF-I:L2	**0.982**	**0.980**	0.0149	78.32 s
jrSiCKLSNMF-I:SH	0.977	**0.980**	0.0180	37.52 s
MOFA+	0.306	0.312	0.112	10.24 s
scAI	0.024	0.015	0.0268	160.93 s
Seurat	0.694	0.710	0.0947	36.87 s
U(1,1.5),N(−0.5,0.5)	Mean	Median	Standard Deviation	Mean Time
BREM-SC	0.047	0.022	0.0934	1,253.69 s
CiteFuse	0.182	0.189	0.0520	**9.23 s**
jrSiCKLSNMF-B:L2	0.372	0.375	0.0945	115.72 s
jrSiCKLSNMF-B:SH	0.310	0.303	0.0821	49.31s
jrSiCKLSNMF-I:L2	0.465	0.467	0.112	123.00s
jrSiCKLSNMF-I:SH	**0.702**	**0.711**	0.0895	43.77 s
MOFA+	0.215	0.220	0.0593	14.34 s
scAI	0.019	0.012	0.0192	165.11 s
Seurat	0.387	0.394	0.0895	34.54 s
U(1,2),N(−1,1)	Mean	Median	Standard Deviation	Mean Time
BREM-SC*	0.017	0.009	0.0235	1,277.76 s
CiteFuse	0.130	0.130	0.0509	**8.10** s
jrSiCKLSNMF-B:L2	0.152	0.145	0.0453	197.28 s
jrSiCKLSNMF-B:SH	0.141	0.134	0.0393	82.47 s
jrSiCKLSNMF-I:L2	0.200	0.199	0.0380	123.22 s
jrSiCKLSNMF-I:SH	**0.295**	**0.278**	0.0943	59.44 s
MOFA+	0.143	0.140	0.0395	24.79 s
scAI	0.018	0.012	0.0194	158.97 s
Seurat	0.204	0.188	0.0751	57.36

*BREM-SC fails to run on the 99^th^ simulated dataset with added noise U(1,2) in the scRNA-seq modality and N(−1,1) in the scATAC-seq modality. The values displayed in this row exclude the 99^th^ simulated dataset.

### 3.5 Real data example

**FIGURE 4:**
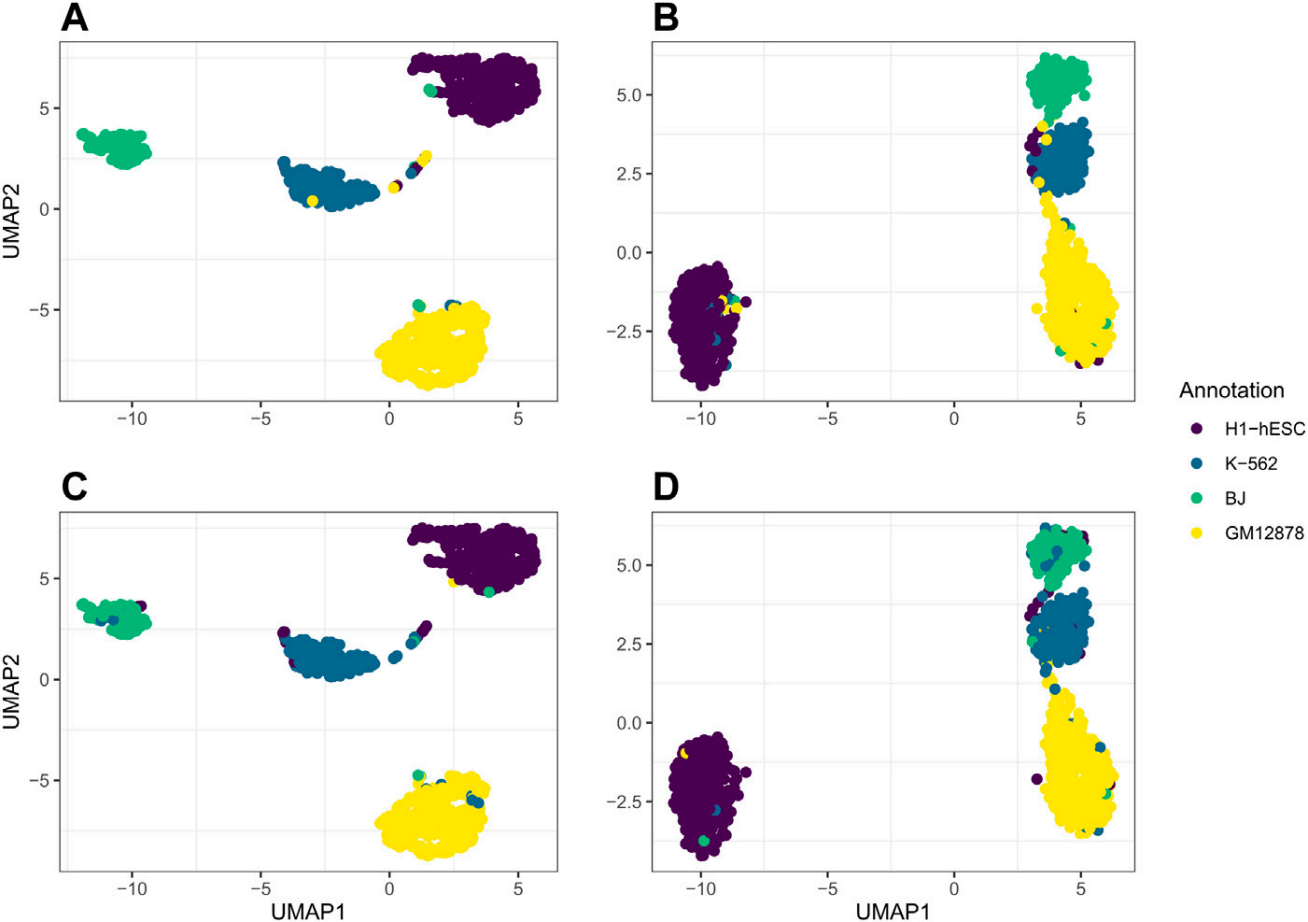
Comparison of UMAP graphs of the H matrix generated by jrSiCKLSNMF with *D* = 10, *λ*W^*RNA*^ = 3, *λ*W^*ATAC*^ = 15, and the unit L2 norm constraint on the rows of **H** **(A,C)** to the Seurat WNN dimension reduction **(B,D)**. The colors of the points in A and B correspond to the generated cell annotations from the RNA modality while the colors of the points in C and D correspond to the ATAC modality.

For our real data example, we use cell line dataset, GSE126074, which includes 1,047 cells from the H1, BJ, K562, and GM12878 cell lines. This dataset is not labeled with the true cell types; however, an R script to generate two sets of cell annotations for the dataset was graciously provided by Professor Song Chen, the first author of the paper describing SNARE-seq (Chen et al., 2019). To annotate the cells, Chen et al. (2019) separately cluster and then annotate the cells in the ATAC modality using cisTopic (Bravo González-Blas et al., 2019) and in the RNA modality using Pagoda2 (Barkas et al., 2021). The ARI between these two annotations was 0.867. We will compare our clustering results to these annotations. Since the data are already pre-processed, we remove 0 cells from the dataset. There are 18,666 genes and 136,771 peaks. We select genes and bins which appear in at least 10 cells and are left with 9,000 genes and 24,514 peaks. In Figure 4, we compare the performance on this dataset of jrSiCKLSNMF with a unit L2 norm constraint on the rows of **H** to the dimension reduction generated by Seurat’s WNN. From these images, we can see that our dimension-reduction method does a better job of separating the cell types into distinct clusters in the UMAP space; one can easily see from this graph that there are 4 clusters. On the other hand, for the Seurat dimension reduction, H1-hESC is clearly separated from the other 3 cell types, but the clusters K-562, BJ, and GM12878 are very close in the UMAP space. Without these color annotations, it could be interpreted as one oblong cluster. Our clustering results are also better. After performing k-means on the H matrix generated here, we achieve an ARI of 0.923 with the annotations based on the RNA modality and 0.916 with the annotations based on the ATAC modality. For the Seurat multimodal WNN analysis, the ARI is 0.876 for the RNA modality and 0.872 for the ATAC modality.

**FIGURE 5:**
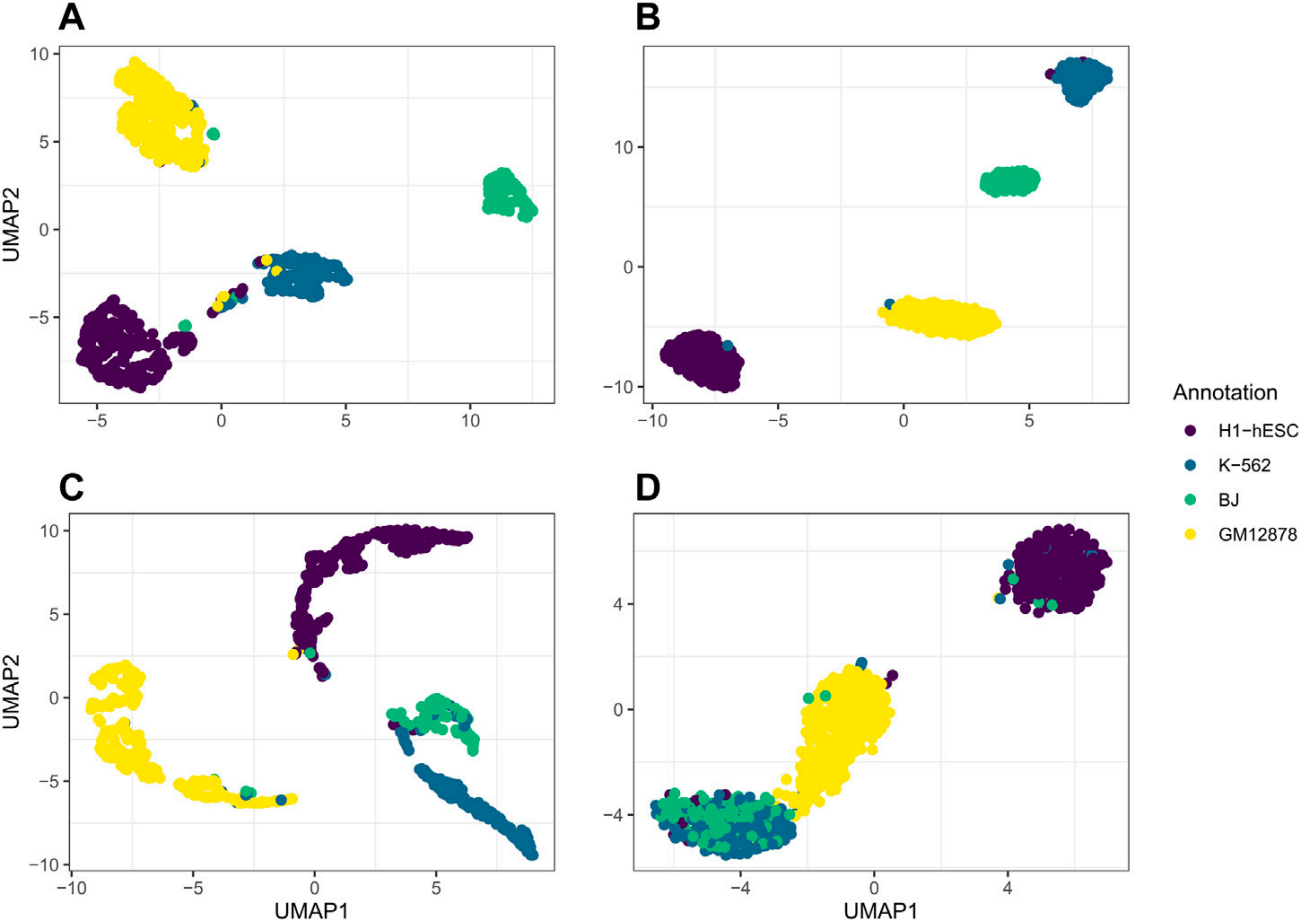
**(A)** is the UMAP of the product of **W** ^**RNA**^ **H** generated by jrSiCKLSNMF with *D* = 10, *λ*W^*RNA*^ = 3, *λ*W^*ATAC*^ = 15, and the unit L2 norm constraint on the rows of **H**, **(B)** is Seurat’s dimension reduction of the RNA modality alone, **(C)** is the UMAP of the product of **W** ^**ATAC**^ **H**, and **(D)** is Seurat’s dimension reduction of the ATAC modality alone. The colors of the annotations for A and B correspond to the generated cell annotations in the RNA modality while the colors of the annotations for C and D correspond to the generated cell annotations in the ATAC modality.

We further use jrSiCKLSNMF to visualize data in the RNA modality and the ATAC modality by performing UMAP on the products **W** ^*RNA*^ **H** and **W** ^*ATAC*^ **H**, respectively. In Figure 5, we plot the UMAP of **W** ^*RNA*^ **H** in (A), the UMAP of **W** ^*ATAC*^ **H** in (C) and compare it to the dimension reduction in Seurat based on the RNA modality alone (B) and the ATAC modality alone (C). The annotations for A and B correspond to the annotations derived purely from the RNA modality while the annotations for (C) and (D) correspond to the annotations derived purely from the ATAC modality. From this, in the first row, we can see that the Seurat UMAP on the RNA dimension reduction almost perfectly captures the four cell types identified by the annotation while our method does not have as clear of a separation in the RNA modality. However, for the ATAC modality, the UMAP of the Seurat dimension reduction fails to capture differences between BJ cells and K-562 cells in the first 2 UMAP dimensions. However, jrSiCKLSNMF is able to capture this difference better: there is a separation between the bulk of the BJ cells and the bulk of the K-562 cells.

**FIGURE 6:**
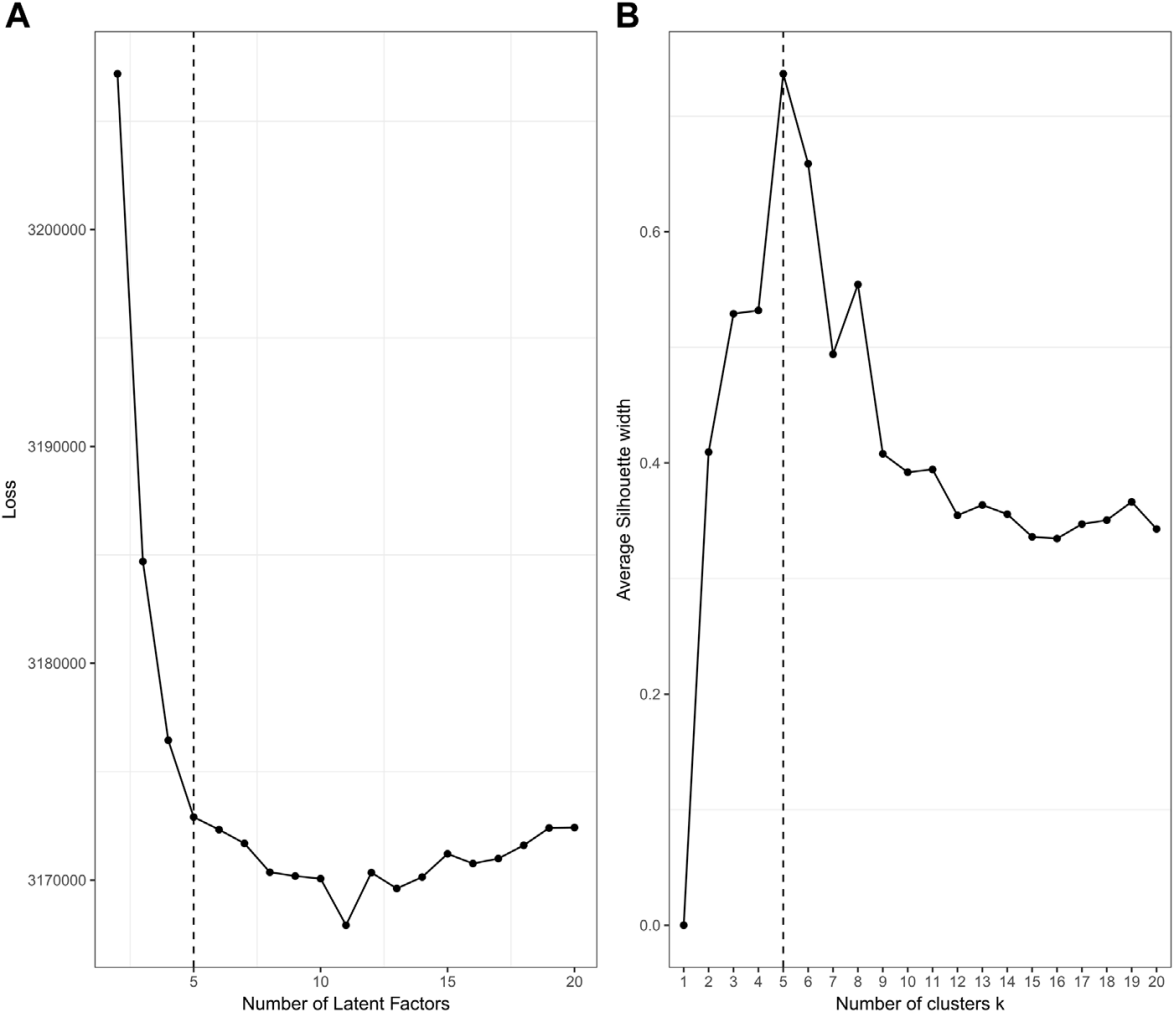
Diagnostic plots for jrSiCKLSNMF to determine the number of latent factors **(A)** and to determine the number of clusters **(B)**, with $\lambda_{W^{\mathrm{RNA}}}=10$ , $\lambda_{W^{\mathrm{ATAC}}}=50$ , and *λ*_*H*_ = 500. In **(A)**, the value of the loss function is after 100 iterations of jrSiCKLSNMF. In **(B)**, we show diagnostics for the silhouette score. Here, the dashed line indicates the ideal number of latent factors and number of clusters, which we determine to be 5 for both of these the number of factors and the number of clusters are coincidentally determined to be equal here. The true number of clusters is 4.

**FIGURE 7:**
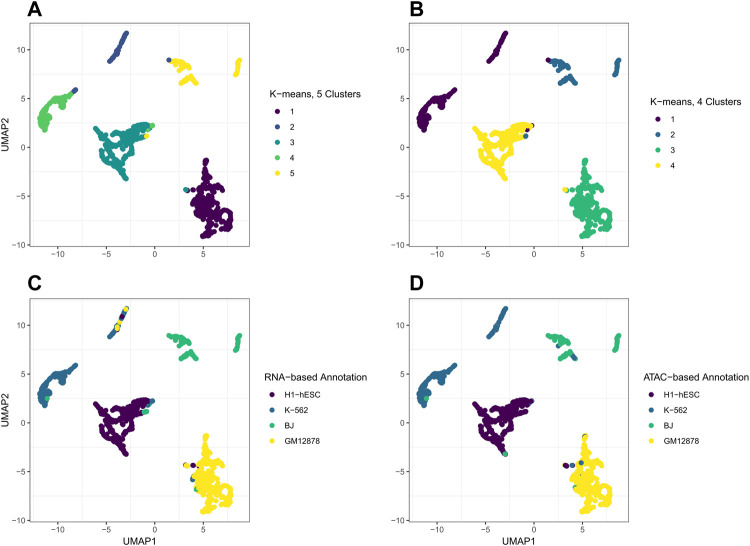
UMAP plots of the **H** matrix that resulted from jrSiCKLSNMF with *D* = 5, $\lambda_{W^{\mathrm{RNA}}}=10$ , $\lambda_{W^{\mathrm{ATAC}}}=50$ , and *λ*_*H*_ = 500, colored by various clustering or annotation results. (**A)** shows the results of clustering **H** using k-means with *k* = 5, as determined by the diagnostic plots in Figure 6. The ARI of these clusters with the RNA annotation is 0.885 and the ATAC annotation is 0.876. (**B)** shows the results of clustering **H** using k-means with *k* = 4, the correct number of cell types. The ARI of these clusters with the RNA annotation is 0.904 and with the ATAC annotation is 0.918. **(C)** plots the UMAP with colors based on the RNA annotations while **(D)** plots the UMAP with colors based on the ATAC annotations.

The plotting performance of jrSiCKLSNMF using the L2 norm constraint is a bit more robust to specifying a larger *D* and obtains slightly better results than jrSiCKLSNMF with a sparsity constraint on **H**. To determine an appropriate number of *D* and *k*, we use diagnostic plots implemented in the jrSiCKLSNMF package. In Figure 6A, for $\lambda_{W^{\mathrm{RNA}}}=10,\lambda_{W^{\mathrm{ATAC}}}=50,\lambda_{H}=500$ we show a plot of the loss function vs. *D* for 2 to 20 factors. We recommend identifying an appropriate elbow. Here, we identify 5 as an appropriate number of factors. After convergence, we perform diagnostics to determine an appropriate number of clusters. In Figure 6B, we provide a representative plot of the silhouette method (the plots using the gap statistic and within sum of squares method are available in the Supplementary Figure S5 while the output from clValid is in Supplementary Table S1). Then, in Figure 7A, we provide a UMAP plot colored by the k-means clusters with number of clusters *k* = 5. In Figure 7B, we provide a UMAP plot colored by clusters determined by k-means using the true number of clusters (4). Figures 7C, D show the RNA and ATAC annotations, respectively. Even though we determined an incorrect true number of clusters, the ARI dropped only from 0.904 to 0.885 in the RNA modality and from 0.918 to 0.875 in the ATAC modality.

## 4 Discussion

jrSiCKLSNMF is a promising method for the analysis of multimodal single-cell count data with many useful properties. First, this method utilizes all features shared across a pre-specified threshold of cells rather than a small subset of highly-variable features. We also do not introduce bias by performing log(*x* + 1) normalization and therefore preserve the count nature of the data in each modality (Townes et al., 2019; Elyanow et al., 2020). This NMF method can provide an intuitive way to summarize and describe data. There is potential for the use of jrSiCKLSNMF in the visualizations of multimodal data because it can extract relevant latent factors from high dimensional data and also provide a method of data compression.

For smaller datasets (i.e., *N* ≪ *M* ^*v*^), we recommend using the I-SH variant of our algorithm. It is not recommended to construct KNN graphs from data where *N* > *M* ^*v*^ or *N* ≈ *M* ^*v*^ because KNN is unreliable in these situations. In this case, we recommend constructing the KNN graph from bulk data or using a graph that is not based on the Euclidean distance. Additionally, when not confident about the number of latent factors, the L2 Norm regularization appears to be slightly more robust to choice of *D* for visualization purposes. Therefore, we recommend using it as a secondary method of data analysis if desired.

Though we show that our method performs well for cell-type clustering, even in the presence of increasing noise, there are a few limitations. These limitations can serve as directions for future research. Firstly, optimizing the choice of *λ* values is not trivial. Through extensive validation, when k-means is used to cluster **H**, we find that for both our simulated data and the real dataset, using *λ*W^*RNA*^ = 10, *λ*W^*ATAC*^ = 50, and *λ*_*H*_ = 500 work well when using a sparsity constraint on **H**, and using *λ*W*^RNA^* = 3 and *λ*W^*ATAC*^ = 15 work well when enforcing a unit norm constraint on the L2 norms of the rows of **H**. However, we also find that, even when using *λ* values that are sub-optimal for clustering using k-means, jrSiCKLSNMF still can extract meaningful factors. We find that for some combinations of *λ* values where k-means performs poorly, the UMAP plot was still accurate, and Louvain clustering performs well.

**FIGURE 8:**
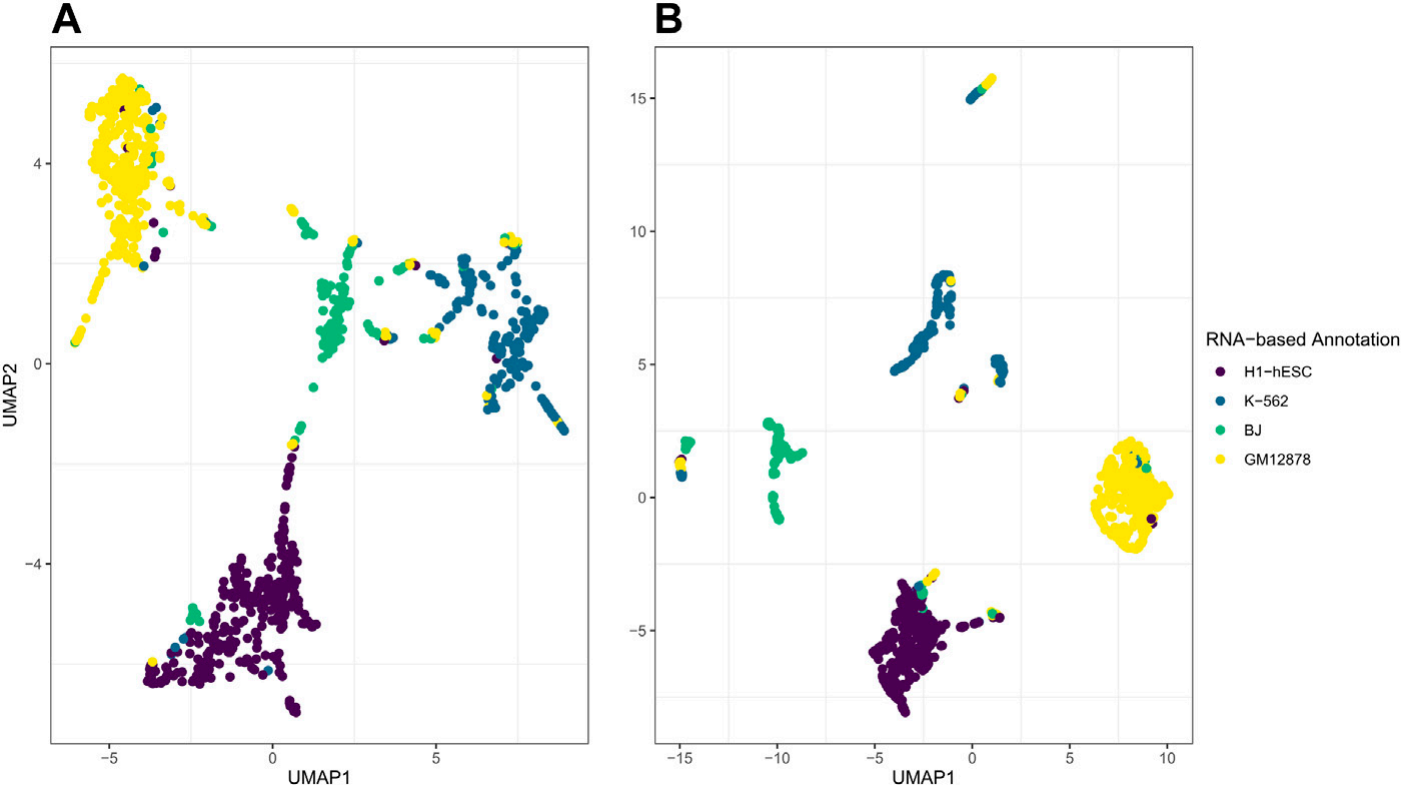
Illustrations of jrSiCKLS-NMF with too much noise captured in the generated **H**. On the left in **(A)** is the UMAP generated from **H** when *D* = 20, $\lambda_{W^{\mathrm{RNA}}}=3,\lambda_{W^{\mathrm{RNA}}}=15$ with the unit L2 norm constraint on the rows of **H**, while on the right in **(B)**, *D* = 10 and $\lambda_{W^{\mathrm{RNA}}}=10,\lambda_{W^{\mathrm{ATAC}}}=50,\lambda_{H}=500$ .

While the *post hoc* clustering remains accurate while varying the number of latent factors *D*, the performance of the visualization using the first two UMAP dimensions depends on appropriate selection of *D*. In Figure 8, on the left in A, we illustrate what happens when the number of latent factors *D* is too large for the variation to be captured within the first 2 elements of UMAP. Here, we set *D* = 20, set $\lambda_{W^{\mathrm{RNA}}}=3$ , $\lambda_{W^{\mathrm{ATAC}}}=15$ , *λ*_*H*_ = 0 and used the unit constraint on the L2 norm of the rows of **H**. In contrast to A and C in Figure 4 where the clusters are well-separated in the UMAP space, here, except for GM12878, the clusters are not as clearly separated. Similarly, in B, which is generated from jrSiCKLSNMF with *D* = 10, $\lambda_{W^{\mathrm{RNA}}}=10$ , $\lambda_{W^{\mathrm{ATAC}}}=50$ , and *λ*_*H*_ = 500, while H1-hESC and GM12878 form distinct clusters, K-562 and BJ appear to form multiple smaller clusters. If we contrast these plots with those in Figures 4, 7, we see that these results capture more noise. Although the plots of the first and second UMAP dimension are not ideal, the clusters determined by using k-means on the respective **H** matrices are still accurate. Optimizing *D* is not trivial and is still an active area of research for NMF (Maisog et al., 2021). While we do provide this method to determine an appropriate *D* visually as in Figure 6A, future research will further address this gap and potentially identify more suitable approaches for the selection of *D*.

Additionally, although our method outperforms existing methods in terms of accurately identifying clusters by a wide margin, the algorithmic implementation can be slower than desirable, especially when we need to determine an appropriate number of latent factors *D* and clusters *k*. Since the methods to determine the number of latent factors *D* and clusters *k* for any of the methods used on simulated data as outlined in Table 2 require pre-specification, for this simulation study, we use a fixed *D* = 10 for our method and the known *k* = 3 for all methods, except for Seurat, which requires a resolution parameter. We therefore fix Seurat’s resolution parameter to a value which consistently results in 3 clusters. Therefore, for these time trials, we do not include the time required to determine the number of clusters for any method or the number of latent factors for our method. For large datasets, this means that it can be computationally demanding to use jrSiCKLSNMF. Although we have implemented sparse matrix functions to decrease memory load and increase speed, methods such as implementing a more efficient descent algorithm than MU, or exploring also using online algorithms as in the 2021 version of LIGER (Gao et al., 2021) may help to improve performance. Moreover, the choice of the KL-divergence itself has some drawbacks. Compared to the wide variety of methods that leverage block coordinate descent to increase the convergence speed of NMF algorithms that use the Frobenius norm, since the KL-divergence is not differentiable for **W** or **H** when (**WH**)_*ij*_ = 0, the KL-divergence lacks the appropriate smoothness requirements to implement block coordinate descent in many cases (Hien and Gillis, 2021). This adds restrictions to the extension of block coordinate descent to KL-NMF algorithms. Hien and Gillis (2021) further discuss that while MU is slow and should not be used in Frobenius NMF algorithms, MU is one of the three most reliable algorithms of the seven descent algorithms for KL-NMF compared in their work. Furthermore, as the technology progresses, datasets will become even larger and will contain more diverse cell types. Testing on a larger number of cell types may have other computational issues. Future works will focus on improving these computational aspects.

Finally, in this work, other than a brief discussion of using **W** ^*v*^ **H** to visualize data in different modalities, we do not address potential applications of the **W** ^*v*^ matrices. Since our focus is on the integration of data from different modalities for the same set of single cells, discussion of applications of **W** ^*v*^ is outside of the scope of this work. **W** ^*v*^ belongs to the feature space rather than the observation space. However, there are many interesting potential avenues for future research involving these **W** ^*v*^ matrices. One such potential application, with which we have had some preliminary success, is using the weighted average of ${\left(Wv\right)}^{+}X_{\mathrm{new}}^{v}$ , where ${\left(W^{v}\right)}^{+}$ is the pseudoinverse of **W** ^*v*^ fitted on the original data **X** ^*v*^ and $X_{\mathrm{new}}^{v}$ is new data, to provide an approximation of **H**_*new*_, the latent factor observation matrix for the new observations. Other such applications include using **W** ^*v*^ to identify co-expressed features or constructing feature networks and exploring whether **W** ^*v*^ **H** can have applications in downstream analyses like network analysis at the single-cell level.

## Data Availability

The original contributions presented in the study are included in the article/Supplementary Material, further inquiries can be directed to the corresponding author.
