# Supplementary material for: Clustering single-cell multimodal omics data with jrSiCKLSNMF
Source: Front Genet. 2023 Jun 9;14:1179439. doi: 10.3389/fgene.2023.1179439 (PMC10288154; doi:10.3389/fgene.2023.1179439)
Supplement: Supplementary file 1 [file DataSheet1.pdf]

Supplementary Material

1 SUPPLEMENTARY TABLES

Method	2	3	4	5	6	7	8	9	10
Connectivity	**4.888**	14.461	24.607	27.213	35.095	53.614	59.738	65.789	143.992
Dunn	**0.106**	0.043	0.040	0.043	0.018	0.022	0.022	0.022	0.022
Silhouette	0.417	0.500	0.532	**0.737**	0.709	0.635	0.599	0.560	0.435

Table S1. Diagnostics from CLVALID (Brock et al., 2008) package for k-means. The connectivity, Dunn index, and silhouette scores are validation metrics to evaluate clustering algorithms. Bold entries indicate the best score. The connectivity and Dunn index both indicate 2 as the optimal number of clusters while the silhouette score indicates 5 as the optimal number.

2 SUPPLEMENTARY FIGURES

All supplementary figures were generated via the R package GGPLOT2 (Wickham, 2016).

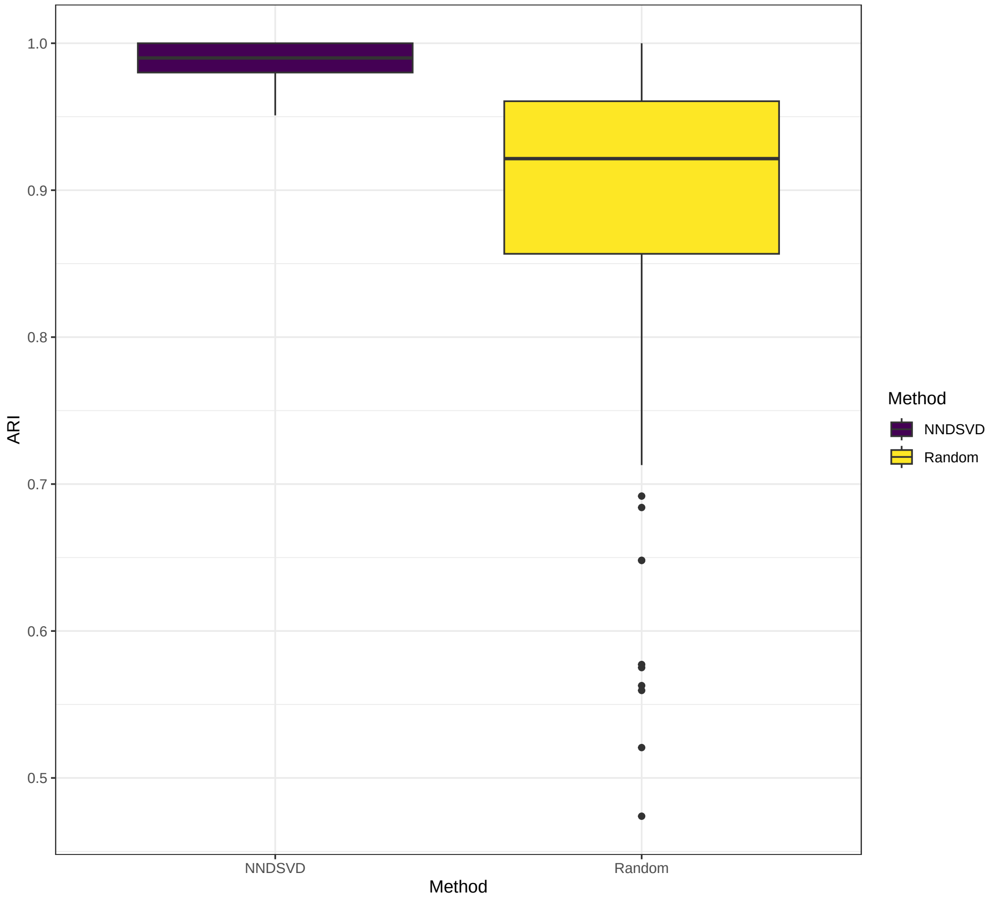

Figure S1. Plot of initialization method (NNDSVD (Qiao, 2015) and random initialization) vs. the achieved ARI with convergence 10^{-6} on simulated data with no added noise and $\lambda_{W^{RNA}} = 10, \lambda_{W^{ATAC}} = 50, \lambda_H = 500$. NNDSVD consistently outperforms random initialization.

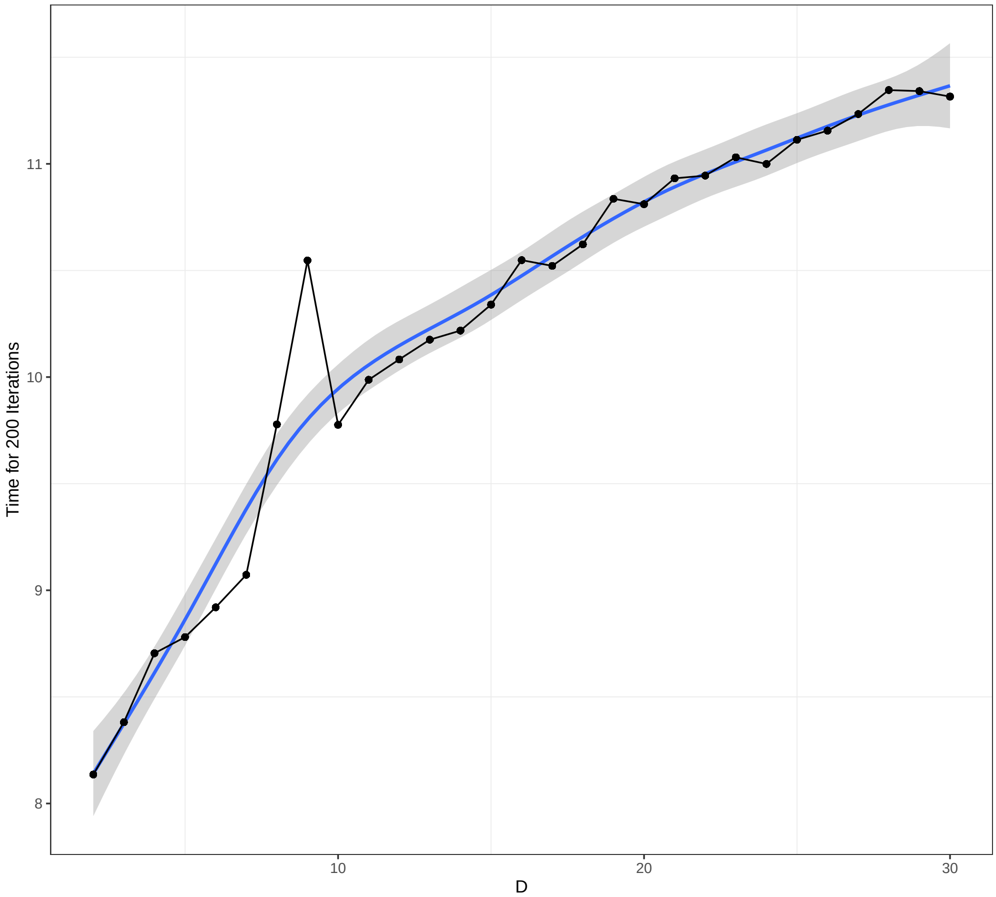

Figure S2. Plot of number of latent factors D, with $D \in 1, ..., 20$ vs the time to complete 200 iterations. The black line is drawn through all of the average points while the blue line indicates a fitted Loess curve. As D increases, the amount of time to complete iterations increases. Please note that the computational times here are substantially higher than normal since this is computed in parallel.

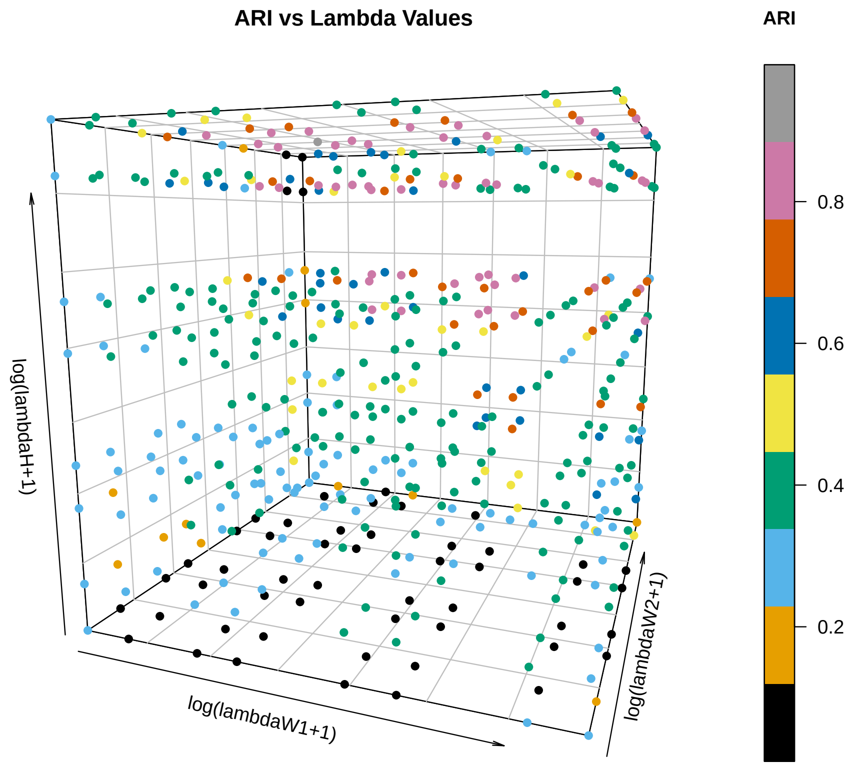

Figure S3. Average ARI for different combinations of $\lambda_{\mathbf{W}^{RNA}}$, $\lambda_{\mathbf{W}^{ATAC}}$, and λ_H for 200 iterations and fixed D on the no-added-noise dataset when computed in parallel on the HiPerGator 3.0 high performance cluster. We run all possible combinations (512) of $\lambda_{\mathbf{W}^{RNA}} = \{0, 1, 5, 10, 50, 100, 500, 1000\}$, $\lambda_{\mathbf{W}^{ATAC}} = \{0, 1, 5, 10, 50, 100, 500, 1000\}$, and $\lambda_H = \{0, 1, 5, 10, 50, 100, 500, 1000\}$. Here the highest average ARI is achieved with $\lambda_{\mathbf{W}^{RNA}} = 5, \lambda_{\mathbf{W}^{ATAC}} = 50, \lambda_H = 1000$ at 0.993. This value is the gray value at the top of the plot toward the left side. We also achieve a high average ARI for $\lambda_{\mathbf{W}^{RNA}} = 10, \lambda_{\mathbf{W}^{ATAC}} = 50, \lambda_H = 500$ of 0.988 and use this for our experiments.

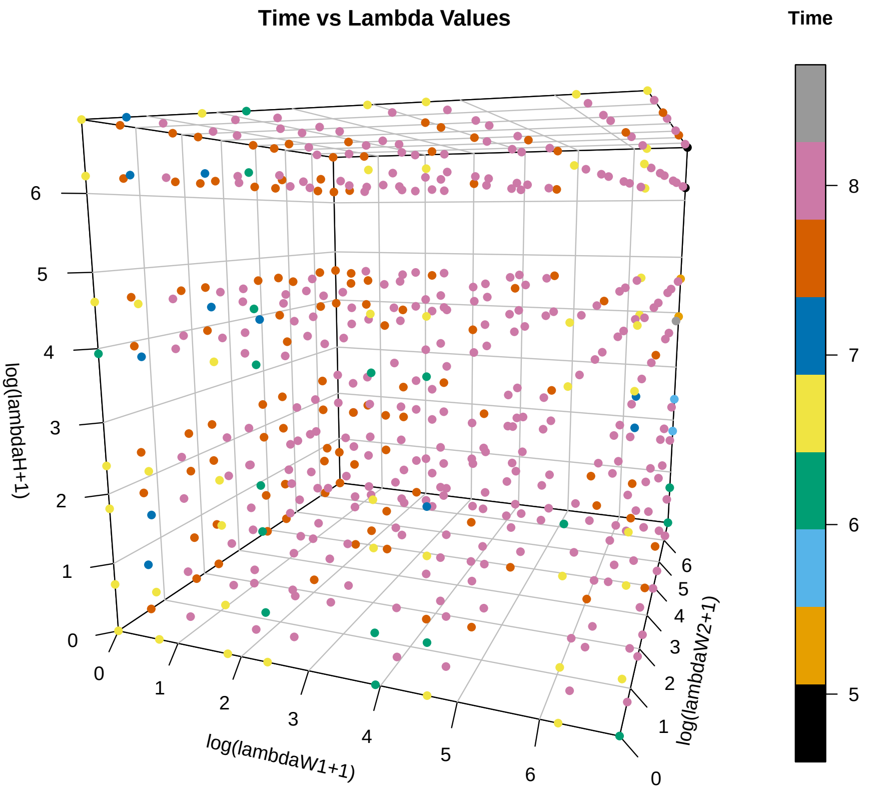

Figure S4. Average time for different combinations of $\lambda_{\mathbf{W}^{RNA}}$, $\lambda_{\mathbf{W}^{ATAC}}$, and λ_H for 200 iterations and fixed D when computed in parallel on the HiPerGator 3.0 high performance cluster. We run all possible combinations (512) of $\lambda_{\mathbf{W}^{RNA}} = \{0, 1, 5, 10, 50, 100, 500, 1000\}$, $\lambda_{\mathbf{W}^{ATAC}} = \{0, 1, 5, 10, 50, 100, 500, 1000\}$, and $\lambda_H = \{0, 1, 5, 10, 50, 100, 500, 1000\}$. The fastest times to convergence (under 6 minutes) are for $\lambda_{\mathbf{W}^{RNA}} = \lambda_{\mathbf{W}^{ATAC}} = 1000$, with $\lambda_H \in (5, 10, 50, 100, 500, 1000)$. Since these are computed in parallel, the time values are inflated due to increased computational overhead when compared to running the algorithm for fewer combinations of $\lambda_{\mathbf{W}^{RNA}}$, $\lambda_{\mathbf{W}^{ATAC}}$, and λ_H.

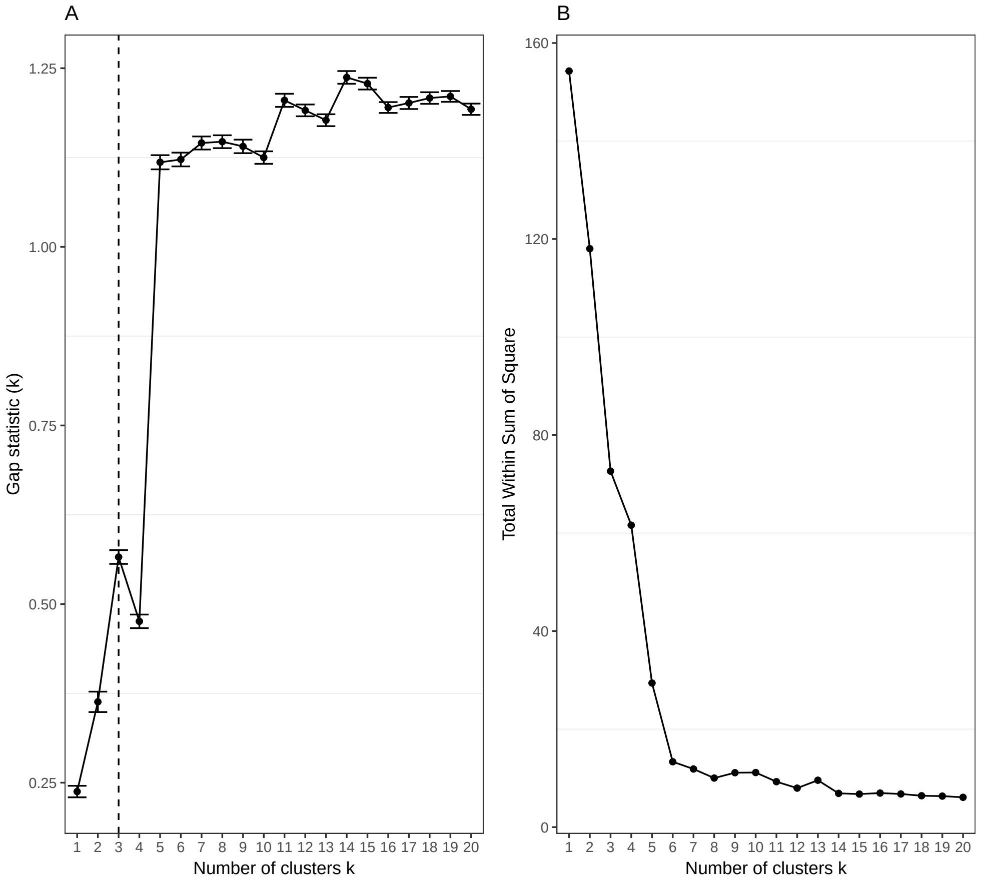

Figure S5. Diagnostic plots to help determine the number of clusters for jrSiCKLSNMF of the gap statistic (A) and the total within sum of square (B). From the gap statistic, 3 was determined to be an ideal number of clusters wile for the total within sum of square method, either 5 or 6 clusters appears valid. The true number of cell types is 4.

REFERENCES

Brock, G., Pihur, V., Datta, S., and Datta, S. (2008). clValid: An R Package for Cluster Validation. *Journal of Statistical Software* 25, 1–22

Qiao, H. (2015). New SVD based initialization strategy for non-negative matrix factorization. *Pattern Recognition Letters* 63, 71–77. doi:10.1016/J.PATREC.2015.05.019

Wickham, H. (2016). *ggplot2: Elegant Graphics for Data Analysis* (Cham, Switzerland: Springer International Publishing), 2 edn. doi:https://doi.org/10.1007/978-3-319-24277-4
